# Effects of Age, Gender, Health Status, and Political Party on COVID-19–Related Concerns and Prevention Behaviors: Results of a Large, Longitudinal Cross-sectional Survey

**DOI:** 10.2196/24277

**Published:** 2021-04-28

**Authors:** Arash Naeim, Ryan Baxter-King, Neil Wenger, Annette L Stanton, Karen Sepucha, Lynn Vavreck

**Affiliations:** 1 Center for SMART Health Departments of Medicine and Bioengineering David Geffen School of Medicine at UCLA and Samueli School of Engineering and Applied Science Los Angeles, CA United States; 2 Department of Political Science College of Letters and Sciences University of California, Los Angeles Los Angeles, CA United States; 3 Division of General Internal Medicine and Health Sciences Research David Geffen School of Medicine at UCLA University of California, Los Angeles Los Angeles, CA United States; 4 Department of Psychology and Psychiatry College of Letters and Sciences University of California, Los Angeles Los Angeles, CA United States; 5 Health Decision Sciences Center Massachusetts General Hospital Harvard Medical School Boston, MA United States; 6 Departments of Political Science and Communication College of Letters and Sciences University of California, Los Angeles Los Angeles, CA United States

**Keywords:** COVID-19, prevention, behavior, advice, health care provider, economy, health information, concern, survey

## Abstract

**Background:**

With conflicting information about COVID-19, the general public may be uncertain about how to proceed in terms of precautionary behavior and decisions about whether to return to activity.

**Objective:**

The aim of this study is to determine the factors associated with COVID-19–related concerns, precautionary behaviors, and willingness to return to activity.

**Methods:**

National survey data were obtained from the Democracy Fund + UCLA Nationscape Project, an ongoing cross-sectional weekly survey. The sample was provided by Lucid, a web-based market research platform. Three outcomes were evaluated: (1) COVID-19–related concerns, (2) precautionary behaviors, and (3) willingness to return to activity. Key independent variables included age, gender, race or ethnicity, education, household income, political party support, religion, news consumption, number of medication prescriptions, perceived COVID-19 status, and timing of peak COVID-19 infections by state.

**Results:**

The data included 125,508 responses from web-based surveys conducted over 20 consecutive weeks during the COVID-19 pandemic (comprising approximately 6250 adults per week), between March 19 and August 5, 2020, approved by the University of California, Los Angeles (UCLA) Institutional Review Board for analysis. A substantial number of participants were not willing to return to activity even after the restrictions were lifted. Weighted multivariate logistic regressions indicated the following groups had different outcomes (all *P*<.001): individuals aged ≥65 years (COVID-19–related concerns: OR 2.05, 95% CI 1.93-2.18; precautionary behaviors: OR 2.38, 95% CI 2.02-2.80; return to activity: OR 0.41, 95% CI 0.37-0.46 vs 18-40 years); men (COVID-19–related concerns: OR 0.73, 95% CI 0.70-0.75; precautionary behaviors: OR 0.74, 95% CI 0.67-0.81; return to activity: OR 2.00, 95% CI 1.88-2.12 vs women); taking ≥4 medications (COVID-19–related concerns: OR 1.47, 95% CI 1.40-1.54; precautionary behaviors: OR 1.36, 95% CI 1.20-1.555; return to activity: OR 0.75, 95% CI 0.69-0.81 vs <3 medications); Republicans (COVID-19–related concerns: OR 0.40, 95% CI 0.38-0.42; precautionary behaviors: OR 0.45, 95% CI 0.40-0.50; return to activity: OR 2.22, 95% CI 2.09-2.36 vs Democrats); and adults who reported having COVID-19 (COVID-19–related concerns: OR 1.24, 95% CI 1.12-1.39; precautionary behaviors: OR 0.65, 95% CI 0.52-0.81; return to activity: OR 3.99, 95% CI 3.48-4.58 vs those who did not).

**Conclusions:**

Participants’ age, party affiliation, and perceived COVID-19 status were strongly associated with their COVID-19–related concerns, precautionary behaviors, and willingness to return to activity. Future studies need to develop and test targeted messaging approaches and consider political partisanship to encourage preventative behaviors and willingness to return to activities.

## Introduction

On January 20, 2020, the first US case of a novel virus was detected [1], and it was later named SARS-COV-2 [2,3]. The coronavirus is spread primarily via exposure to oral and nasal secretions when a person is in close contact with someone who has COVID-19 [4], and it is known to cause critical illness and substantial mortality in a subset of the infected population [5]. As of August 27, 2020, a total of 24,234,340 confirmed cases and 827,110 deaths due to COVID-19 were reported worldwide [6], of which over 5,752,653 cases and 177,759 deaths were reported in the United States alone [7]. These high numbers are in part because SARS-COV-2 is highly contagious [8], and about 44% (95% CI 30%-57%) of the confirmed cases are due to presymptomatic transmission [9-11].

Without a vaccine or an effective treatment, the COVID-19 pandemic could easily overwhelm hospitals. Actions undertaken at the individual level, such as washing hands, social distancing [12], and wearing face masks [13], can slow the spread of the disease [14]. Government measures to curtail the spread started with international travel restrictions [15]. California was the first to issue state-wide stay-at-home orders [16], followed by most, but not all (8 states did not), states [17]. The states with stay-at-home orders successfully reduced the contagion compared to states that did not enforce these orders [18]. Stay-at-home policies have significant economic and social consequences [19]; federal, state, and local officials now struggle to protect American lives while also recovering the economy by enabling people to go back to work [20].

Multiple factors influence COVID-19 precautionary and return-to-activity behaviors. For example, knowledge about COVID-19 and its risks are associated with a high-risk behavior, such as attending large gatherings and not wearing masks [21]. Additionally, the risk of getting severely ill from COVID-19 increases with age and coexisting conditions [22], and the risk is higher in men than in women [23,24]. Among some racial and ethnic groups in specific contexts, evidence points to higher rates of hospitalization and death due to COVID-19, and this is especially true in vulnerable populations such as migrants and undocumented individuals [25-27]. Political orientation and environment also are associated with COVID-19 response recommendations [21,28]. Finally, income disparities can result in situations that pit physical distancing against meeting basic needs [29].

With conflicting statements in the media from medical and political leaders, the general public lives with considerable uncertainty about the disease and the impact of their behaviors on their health and the health of the community. Understanding the factors affecting adults’ willingness to engage in precautionary behavior or to return to normal activities could improve messaging among those with trusted voices and potentially aid economic recovery [30]. Using cross-sectional, national, population-based surveys conducted across time, this exploratory study examines major factors associated with COVID-19 precautionary behaviors, willingness to return to typical activities when told it is safe to do so by public health officials, and the levels of concern about the virus in order to inform preventive efforts and determine key populations that may benefit from reinforced messaging from health care providers. Our hypothesis was that the identification of political party affiliation would have as large an effect as other traditional covariates such as age, gender, race or ethnicity, health (ie, number of prescriptions), education, information, income, COVID-19 status, and religion with regard to three key outcomes: (1) COVID-19–related concerns, (2) precautionary behaviors, and (3) willingness to return to activity.

## Methods

### The Democracy Fund + UCLA Nationscape Project

Data were obtained from 20 weeks of the ongoing Democracy Fund + UCLA Nationscape Project, a weekly survey comprising 6250 people ([31], Multimedia Appendix 1). The sample was provided by Lucid, a market research platform. Web-based surveys were administered. UCLA staff set quotas for sample acquisition and generated weights to produce a nationally representative sample of the adult American population. Additional information on the survey methodology and the data’s comparability to population targets are available [31]. Nationscape is well suited to examine the impact of COVID-19 due to its size and geographic scope. The wording of questions and response options are available on the internet [32]. This project was approved by the University of California, Los Angeles (UCLA) Institutional Review Board (IRB #19-000897).

### Key Outcome Variables

Three outcomes were considered: (1) COVID-19–related concerns, (2) precautionary behaviors, and (3) willingness to return to activity. Not all outcomes were asked in each survey wave; therefore, the sample size varies among outcomes.

#### COVID-19–Related Concerns

Concerns about COVID-19 were coded as four categories: “Not at all concerned,” “Not very concerned,” “Somewhat concerned,” and “Very concerned.”

#### Precautionary Behaviors

Precautionary behaviors included individual items on washing hands, wearing a face mask, limiting visits to family members, quarantining oneself, and cancelling travel plans. Respondents were asked whether they had performed each of these activities in response to the spread of COVID-19 (response options: yes or no). In surveys conducted after May 28, 2020, respondents were specifically asked whether they had worn a face mask when going out in public within the last week. The return to activity component evaluated the respondents’ routine activities, including having dinner with friends, attending a funeral, attending a wedding, attending church, getting a haircut, visiting a dentist, going to a shopping mall, sending a child to school, going to school oneself, taking a flight, going to the movies, using public transit, attending a sporting event, and attending a concert. Respondents were asked whether they would return to a given activity if the restrictions on doing so were lifted on the advice of public health officials (response options: yes or no). Individuals who reported that they “would not have done this activity before the COVID-19 pandemic” for a given activity were excluded from further analyses.

#### Key Independent Variables

Key independent variables included age (age range: 18-39, 40-64, and ≥65 years), gender (male or female), race or ethnicity (Hispanic, White, Black, Asian or Pacific Islander, or other), education (high school or lower, some college, and college and beyond), household income (by tercile), political party they support (Democrat, Independent, or Republican), religion (Protestant, Catholic, Jewish, Mormon, other, or not religious), evangelical (evangelical or not evangelical), political news consumption (0-2, 3-6, or ≥7 news sources), number of prescription medications (0-3 or ≥4), COVID-19 status (believes they had COVID-19 or not), and the timing of COVID-19 infections by state (early peak, later peak, or no peak or low rate). For the last variable, “early peak” states were those that reached a threshold of 30 confirmed cases per 100,000 residents before June 1, 2020; “later peak” states were those that failed to reach this threshold by June 1, 2020, but eventually reached at least 10 cases per 100,000.

### Data Analysis

The data included 125,508 interviews conducted between March 19 and August 5, 2020. Across the different survey waves, of those respondents selected to be interviewed, 13% (22,115/174,690) declined immediately, and 10% (17,517/174,690) dropped off elsewhere during the survey without completing it. An additional 5% (9550/174,690) were removed after quality control checks. This results in a response yield of 72% (125,508/174,690) of the initially invited sample. Most sample proportions were within a few points of the target population before weights were applied (Table 1, Multimedia Appendix 2). The data were weighted to age, race, ethnicity, gender, education, partisanship, income, and region, among other items; thus, any differences observed here were further minimized after weighting.

Weighted proportions were calculated with R statistical software (version 3.6.1) based on data collected from March to August 2020. Weighted multivariate logistic regression analyses were used to calculate odds ratios (ORs). The survey wave was included as a fixed effect. Additionally, weighted difference-in-means tests assessed whether the trends shown in Multimedia Appendix 3 were statistically significant. These tests compared weighted proportions from the April 16, 2020 wave to the June 11, 2020 wave—both overall and for each subgroup. The reference categories are described in Multimedia Appendix 4. To demonstrate the effect of gender, partisanship, and having contracted COVID-19, the average probabilities of the population engaging in each dependent variable were calculated as if the respondents were all either men or women, Republicans or Democrats, and sick or not sick (leaving other characteristics unchanged) [33]. The difference between the probabilities when everyone was assigned the propensity of men compared to women, for example, illustrates the differences in likelihood due to gender.

**Table 1:** Unweighted and weighted characteristics of the sample population (N=125,508).

Characteristics		Unweighted, n (%)	Weighted (%)	
**Age (years)**				
	18-39	52,413 (41.8)		37.7
	40-64	54,330 (43.3)		41.8
	65+	18,768 (15.0)		20.5
**Gender**				
	Female	66,918 (53.3)		51.7
	Male	58,590 (46.7)		48.3
**Race or ethnicity**				
	Asian or Pacific Islander	8,913 (7.1)		8.0
	Black	12,513 (10.0)		11.2
	Hispanic	17,835 (14.2)		15.5
	Some other race	2,352 (1.9)		1.9
	White	83,898 (66.8)		63.4
**Education**				
	High school or lower	31,983 (25.5)		32.3
	Some college	42,723 (34.0)		36.9
	College and above	50,805 (40.5)		30.9
**Income**				
	1st tercile (US $0-34,999)	42,048 (33.5)		20.3
	2nd tercile (US $34,999-79,999)	44,655 (35.6)		35.5
	3rd tercile (≥US $79,999)	38,808 (30.9)		44.2
**State-level COVID-19 trend**				
	Early peak state	24,939 (19.9)		19.7
	Late peak state	93,516 (74.7)		75.1
	Low rate	6,663 (5.3)		5.2
**Prescriptions**				
	0-3	47,076 (79.6)		79.4
	≥4	12,090 (20.4)		20.6
**Perceived as having contracted COVID-19**				
	Self	8,106 (6.5)		5.2
	Family	10,080 (8.1)		7.5
	Work	114,300 (15.1)		14.0
	Other	105,369 (32.4)		32.0
**Political party support**				
	Democrat	55,947 (44.6)		44.9
	Independent	18,561 (14.8)		16.9
	Republican	50,856 (40.6)		38.1
**News from Facebook**				
	No	35,244 (28.1)		31.9
	Yes	90,267 (71.9)		68.1
**News sources**				
	0-2	31,254 (24.9)		25.9
	3-6	68,286 (54.4)		55.2
	≥7	25,971 (20.7)		18.9

## Results

The data included responses from 125,508 interviews conducted between March 19 and August 5, 2020, approved by the UCLA Institutional Review Board for analyses. Unless otherwise noted, all ORs presented were significant at *P*<.001, with 95% CIs presented.

### COVID-19–Related Concerns

About 57.3% (unweighted: 69,556/122,798) of the respondents were “very” concerned (vs “somewhat,” “a little,” or “not at all” concerned) about COVID-19. Groups more likely to be *very concerned* about COVID-19 were those involving participants aged ≥65 years (OR 2.05, 95% CI 1.93-2.18 vs those aged 18-40 years); Asian or Pacific Islander (OR 1.48, 95% CI 1.38-1.59), Black (OR 1.33, 95% CI 1.25-1.42), or Hispanic participants (OR 1.29, 95% CI 1.22-1.36) versus White participants; participants with college education (OR 1.15, 95% CI 1.1-1.21 vs those with high-school education or lower); participants who took ≥4 medications (OR 1.47, 95% CI 1.40-1.54 vs those who took <3 medications); participants who thought they had contracted COVID-19 (OR 1.24, 95% CI 1.12-1.39 vs those who did not think they had contracted COVID-19); and participants who received news from ≥7 sources (OR 2.37, 95% CI 2.23-2.52 vs those who received news from 0-2 sources). Groups less likely to be *very concerned* about COVID-19 were men (OR 0.73, 95% CI 0.70-0.75 vs women), Independents (OR 0.54, 95% CI 0.51-0.57 vs Democrats), Republicans (OR 0.40, 95% CI 0.38-0.42 vs Democrats), and those who lived in later-peak states (OR 0.83, 95% CI 0.79-0.87 vs those who lived in early-peak states). More detailed results are available in Multimedia Appendix 5.

### Precautionary Behaviors

**Figure 1:**
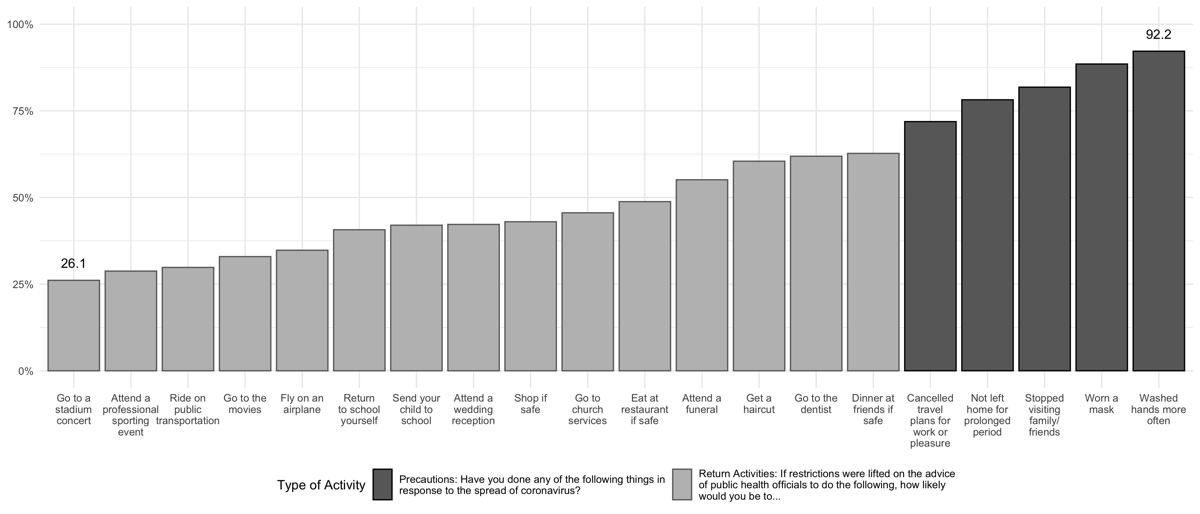
Percentage of the sample population that has undertaken precautions and will return to activities.

**Figure 2:**
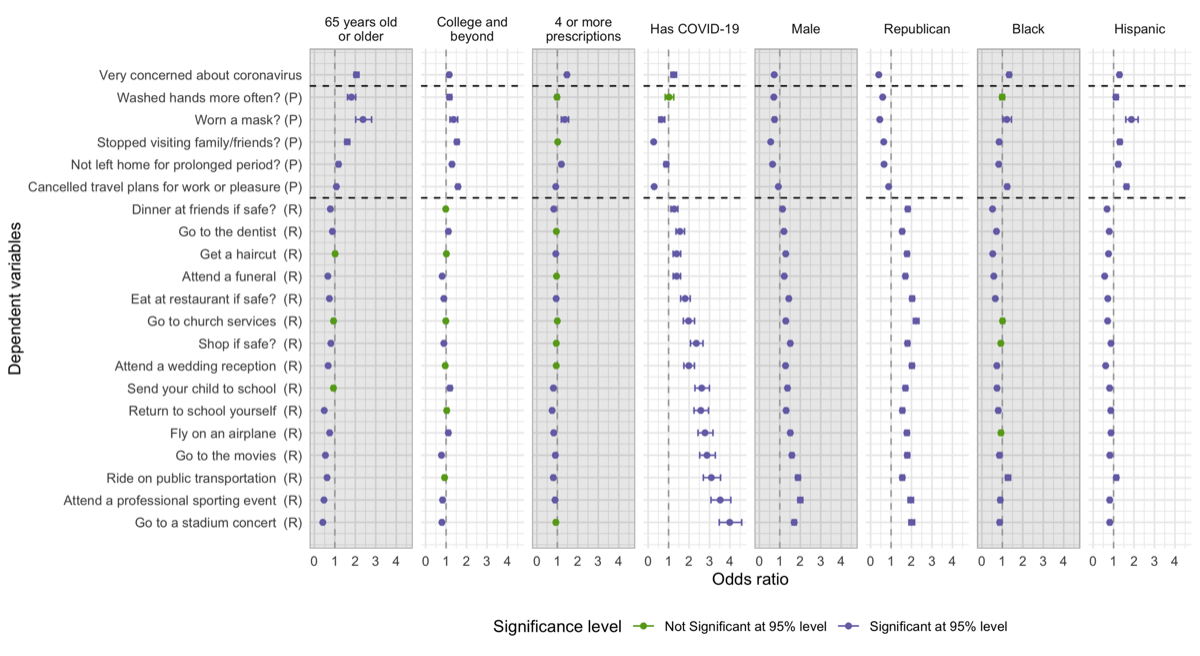
Key predictors of COVID-19–related concerns, taking precautions, and returning to activities. Results control for variables in Multimedia Appendix 4. Unless noted, all controls are at median values. For dependent variables (y-axis), "P" indicates "precaution" and "R" indicates "return activity." Sample size and date range vary by model, see Multimedia Appendix 5 for details. Data are from The Democracy Fund + UCLA Nationscape Project [31].

The majority of individuals (>70%) reported engaging in precautionary behaviors, ranging from 71.9% (unweighted: 43,646/61,844) to 92.2% (unweighted: 56,820/61,987) depending on the specific behavior (Figure 1). For example, the following groups were more likely to wear a face mask (Figures 1 and 2 and Multimedia Appendix 5): older (≥65 years) individuals (OR 2.38, 95% CI 2.02-2.80 vs 18-40 years); Asian or Pacific Islander (OR 2.10, 95% CI 1.71-2.58), Black (OR 1.22, 95% CI 1.03-1.44), and Hispanic participants (OR 1.87, 95% CI 1.60-2.20) versus White participants; those taking ≥4 medications (OR 1.36, 95% CI 1.20-1.55 vs <4 medications); those receiving political news from a greater number (≥7) of sources (OR 2.06, 95% CI 1.75-2.43 vs ≤2 sources), and those with household incomes over US $80,000 annually (OR 1.51, 95% CI 1.31-1.71 vs those with incomes less than $40,000 annually).

The following groups were less likely to wear a mask: men (OR 0.74, 95% CI 0.67-0.81] vs women); people who believe they have had COVID-19 (OR 0.65, 95% CI 0.52-0.81 vs those who do not believe so); participants in late-peak states (OR 0.64, 95% CI 0.55-0.73 vs those in early-peak states); Independents (OR 0.50, 95% CI 0.43-0.57 vs Democrats); or Republicans (OR 0.45, 95% CI 0.40-0.50 vs Democrats).

### Willingness to Return to Activities

A large number of participants would not be willing to return to activities they engaged in before COVID, ranging from 26.1% (unweighted: 15,082/51,773) to 62.7% (unweighted: 37,471/58,504), even after the restrictions are lifted and public health officials declare it is safe to return to such activities (Figure 1). The following groups tended to be less likely to report willingness to return to activities across the board (Figures 1 and 2 and Multimedia Appendix 5): older (≥65 years) individuals (OR 0.41, 95% CI 0.37-0.46 to OR 0.93, 95% CI 0.88-0.98 vs 18-40 years); Asian or Pacific Islander (OR 0.52, 95% CI 0.46-0.59 to OR 0.88, 95% CI 0.80-0.96); Black (OR 0.52, 95% CI 0.48-0.57 to OR 0.90, 95% CI 0.81-1.0), and Hispanic participants (OR 0.56, 95% CI 0.53-0.61 to OR 0.87, 95% CI 0.81-0.95) compared with White participants, except in the use of public transportation among Black participants (OR 1.27, 95% CI 1.16-1.40 vs White participants) and Hispanic participants (OR 1.13, 95% CI 1.04-1.23; *P*<.01 vs White participants); and those taking 4 or more medications (OR 0.75, 95% CI 0.69-0.81 to OR 0.94, 95% CI 0.89-1.00 vs those taking <4 medications).

Respondents who were more likely to report willingness to return to activities included those who believe they had contracted COVID-19 (OR 1.27, 95% CI 1.11-1.45 to OR 3.99, 95% CI 3.48-4.58 vs those who believe they did not); men (OR 1.13, 95% CI 1.08-1.19 to OR 2.00, 95% CI 1.88-2.12 vs women); and Republicans (OR 1.55, 95% CI 1.46-1.63 to OR 2.22, 95% CI 2.09-2.36 vs Democrats). Those who are highly educated (college education or beyond) were significantly less likely to consider participating in activities such as going to the movies, concert, or sporting event (OR 0.77, 95% CI 0.72-0.83 to OR 0.83, 95% CI 0.76-0.90 vs those who have less than or up to high school–level education), but they were more likely to send their children to school (OR 1.19, 95% CI 1.11-1.28), visit a dentist (OR 1.11, 95% CI 1.04-1.19), or travel by air (OR 1.11, 95% CI 1.03-1.19).

### Longitudinal Analysis

**Figure 3:**
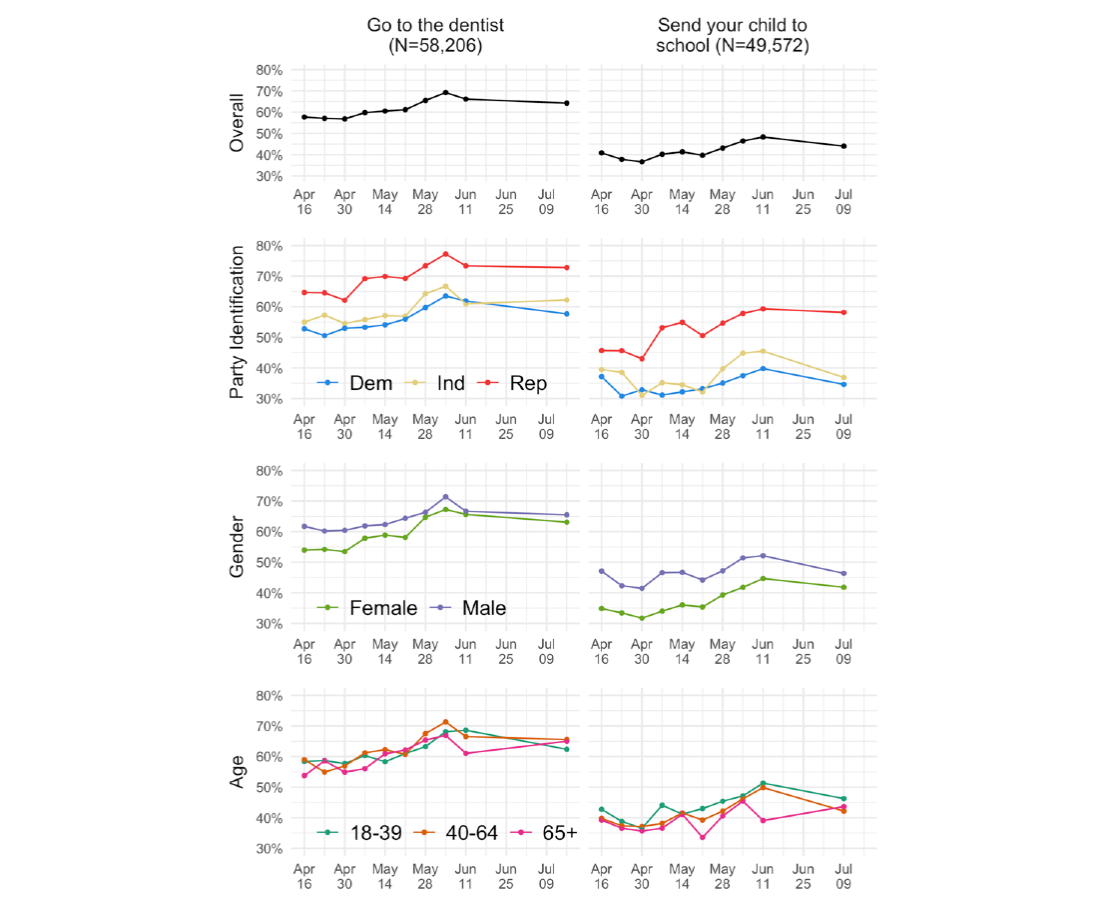
Longitudinal data for two recovery behaviors (going to the dentist and sending your child to school) overall and by political party affiliation, gender, and age.

We conducted a series of weighted difference-in-means tests assessing whether the trends illustrated in Figure 3 changed over time. Specifically, we compared the percentage from the survey wave of April 16, 2020, to that from the survey wave of June 11, 2020. This was the final wave where all outcome variables were collected. These tests were run both for the overall trends for going to the dentist and sending your child to school as well as for each of the subgroups presented in Figure 3. The trends were found to be generally statistically significant (see longitudinal analysis results shown in Multimedia Appendix 3). More people were willing to return to these activities every week between April and June 2020. In June and July, however, the increases generally tapered off, as shown in Figure 3. The rate of increases was similar across gender and age groups, although there was a more pronounced separation noted between Democrats (who were less willing) and Republicans (who were more willing) in July than in April, suggesting a slower rate of increase to return to activities for Democrats, particularly with respect to sending a child to school.

### Predicted Probabilities for Specific Groups

**Figure 4:**
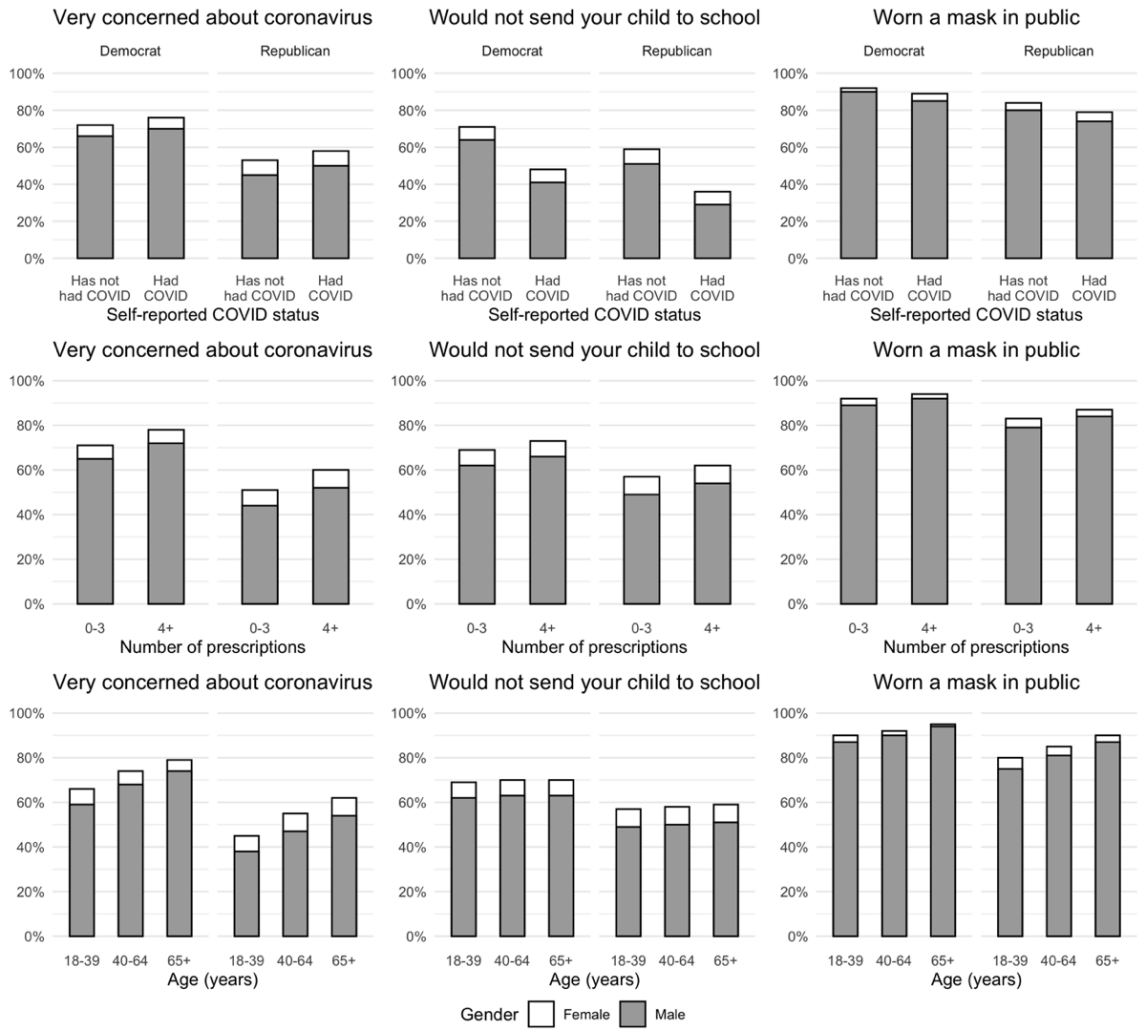
Predicted probabilities of outcomes for key groups.

Several patterns appeared in these analyses. The most concerned group of people were older adults, women, Democrats (with an 80% chance of being concerned), as shown in Figure 4. To understand how age, gender, and political party affiliation come together to shape a person’s orientation toward COVID-19, we compared this group to younger, Republican men, whose chances of being concerned about COVID-19 were found to be the lowest, just under 40%. A similar pattern was observed for sending a child to school and for wearing a mask. Democratic women who took 4 or more medications were 30 points more likely to keep their child home and not send them to school than Republican men who took fewer medications. Similarly, Republicans who thought they have had COVID-19 were 30 points less likely to wear a mask in public than Democrats who did not think they had contracted the infection.

## Discussion

The data from our analyses provide considerable evidence that the majority of Americans have rapidly adopted new preventive behaviors in the face of the deadly COVID-19 pandemic. On average, more than 8 in 10 report wearing a mask outside (unweighted: 70,802/80,624), and most have avoided meeting family and cancelled travel plans. In addition, these data shed light on the challenges that cities and states face as society reopens. Even after public health officials declare activities to be safe to engage in, more than half the survey respondents reported that they will not send a child to school or travel by air, and only 61.9% will visit a dentist (unweighted: 36,540 /58,206). It is unclear whether projected caution is related to the inconsistent messaging or the source of information requires greater exploration than we can provide here and needs to be further evaluated. This may also be related to public uncertainty and trust in the messenger or the message itself [34]. There is significant politicization that casts doubt on public health warnings and in the accuracy of statements from public officials in the recommendations associated with preventive behaviors and how to ramp-up the economy [35,36]. Politicization of COVID-19 preventive behaviors is not limited to the United States, but it is also observed in other countries such as the United Kingdom and Brazil [37,38]. Confusion and mixed messaging (alongside variations in protocols implemented by 50 state governors) calls on the physician to provide guidance that is based on science and perceived as trustworthy by patients [39,40].

Despite the generally high rates of reported precautionary behaviors, the 10%-20% nonadherence rate may exceed the thresholds needed to quell the virus spread [13,41-43]. It is also noteworthy that these rates are likely high estimates because our survey questions asked about whether the behavior was practiced at all, but not how often or how consistently. It should alarm health care providers that small, but possibly substantial, groups of patients are not following public health rules (which may have limited evidence-based data), suggesting that behavioral intention concerning the contagion should be explored with patients. Several studies have shown that level of concern and risk perception is linked to adoption of precautionary behaviors [44-46], which is augmented by varying infection fatality rates [47]. Reinforcing preventive messages is particularly needed for patients who have recovered from COVID-19 infection. Such individuals are less likely to engage in precautionary behaviors, which may be the reason they contracted the virus. It is likely, however, that recovered patients also have developed some immunity against reinfection. This demonstrates the risk of inadequate testing, false-positive serological testing, and assumptions around T-cell immunity in creating a false sense of security that in turn encourages permissive behavior [48-50].

The analyses identified clear patterns in the levels of concern, precautionary behaviors, and willingness to resume activities after the restrictions are lifted. Despite controlling for all other factors, party affiliation was a significant factor for being *very concerned* about COVID-19, engaging in preventive measures, and returning to activities, to an extent not observed in other major disease outbreaks or prior pandemics [51-55]. The group most likely to be concerned about COVID-19 (ie, older adults, female participants, and Democrats) was also more likely to engage in preventive behaviors and less willing to resume activities. A second group (ie, younger adults, male participants, less well-educated participants, and Republicans) reported lower levels of concern about COVID-19, were less likely to report precautionary behaviors, and more willing to return to activities. Previous studies have suggested that people trust medical experts more than political leaders [56,57]. However, it would be short-sighted to neglect how the lens of political party affiliation informs attitudes and how patients process information given the prior evidence that political party influences health domains such as obesity, end-of-life management, and vaccine adoption [58]. Our findings are consistent with those of other studies that highlight the political polarization of preventive health behavior with regard to COVID-19 and in general [21,28,59,60].

This study’s limitations deserve mention. First, this study focused primarily on readily observable factors. However, such factors (eg, gender) are not explanatory by themselves. Relatedly, most health behaviors involve multiple determinants, and the determinants evince substantial interindividual variability. Reliable mask-wearing might result from concern for others’ health, risk aversion, respect for the relevant science, high motivation to comply with rules, among other factors. Evidence for the malleability of risk perceptions, prosocial motivation, and other contributors to health-promoting behaviors point toward promising targets for change [61-64]. Our research group and others [65] are working to identify such malleable targets. The present survey did not collect data on occupation and work-related subsidies. Second, the cross-sectional nature of the data does not allow for definitive interpretation of findings regarding across-time stability and change; however, the sampling strategy and large sample lend confidence to the findings. Third, measures of precautionary behaviors were author-constructed in a way that may overestimate their extent. Fourth, the questions about COVID-19 status did not differentiate between those individuals who had symptoms versus a confirmed laboratory test, yet the construct of believing that one has had COVID-19 is clinically relevant. Finally, in this study, we could not compare the sample population to an international sample to analyze the effect of the prosperity of a country or differences in national health care insurance plans [66,67].

Health care providers have a significant role to play both in managing the pandemic and ensuring adherence to prevention and recovery behaviors. This implies not only making masks mandatory in clinical settings but also strongly counseling patients to wear face coverings [68] in high-risk environments and avoiding high-risk activities. For providers and public health officials to serve as facilitators, they need to understand the attitudes and perceptions of their patients and tailor messages to move them toward both prevention and recovery. This is critical because recovery represents a set of behaviors that impact not just economic health but also the personal health of patients, many of whom have also been deferring the care of their chronic medical illnesses as well as routine but important health maintenance and prevention. Future studies need to develop and test targeted messaging approaches, including those with respect to political party, to encourage preventative behaviors and willingness to return to activities.

## Appendix

Multimedia Appendix 1 CHERRIES (Checklist for Reporting Results of Internet E-Surveys) checklist.

Multimedia Appendix 2 Representativeness table.

Multimedia Appendix 3 Longitudinal analysis of data shown in Figure 2: Difference in means over time for willingness to send children to school and visit the dentist.

Multimedia Appendix 4 Median values for regression analysis.

Multimedia Appendix 5 Regression tables.

## Abbreviations

OR: odds ratio
UCLA: University of California, Los Angeles

## References

[1] Lu R, Zhao X, Li J, Niu P, Yang B, Wu H, Wang W, Song H, Huang B, Zhu N, Bi Y, Ma X, Zhan F, Wang L, Hu T, Zhou H, Hu Z, Zhou W, Zhao L, Chen J, Meng Y, Wang J, Lin Y, Yuan J, Xie Z, Ma J, Liu WJ, Wang D, Xu W, Holmes EC, Gao GF, Wu G, Chen W, Shi W, Tan W (2020). Genomic characterisation and epidemiology of 2019 novel coronavirus: implications for virus origins and receptor binding. Lancet.

[2] Coronaviridae Study Group of the International Committee on Taxonomy of Viruses (2020). The species severe acute respiratory syndrome-related coronavirus: classifying 2019-nCoV and naming it SARS-CoV-2. Nat Microbiol.

[3] Holshue ML, DeBolt C, Lindquist S, Lofy KH, Wiesman J, Bruce H, Spitters C, Ericson K, Wilkerson S, Tural A, Diaz G, Cohn A, Fox L, Patel A, Gerber SI, Kim L, Tong S, Lu X, Lindstrom S, Pallansch MA, Weldon WC, Biggs HM, Uyeki TM, Pillai SK, Washington State 2019-nCoV Case Investigation Team (2020). First case of 2019 novel coronavirus in the United States. N Engl J Med.

[4] How COVID-19 Spreads. Centers for Disease Control and Prevention.

[5] Cummings MJ, Baldwin MR, Abrams D, Jacobson SD, Meyer BJ, Balough EM, Aaron JG, Claassen J, Rabbani LE, Hastie J, Hochman BR, Salazar-Schicchi J, Yip NH, Brodie D, O'Donnell MR (2020). Epidemiology, clinical course, and outcomes of critically ill adults with COVID-19 in New York City: a prospective cohort study. The Lancet.

[6] COVID-19 Dashboard by the Center for Systems Science and Engineering (CSSE) at Johns Hopkins University (JHU). Johns Hopkins University & Medicine - Coronavirus Resource Center.

[7] United States COVID-19 Cases and Deaths by State. Centers for Disease Control and Prevention.

[8] Sanche S, Lin YT, Xu C, Romero-Severson E, Hengartner N, Ke R (2020). High contagiousness and rapid spread of severe acute respiratory syndrome coronavirus 2. Emerg Infect Dis.

[9] He X, Lau EHY, Wu P, Deng X, Wang J, Hao X, Lau YC, Wong JY, Guan Y, Tan X, Mo X, Chen Y, Liao B, Chen W, Hu F, Zhang Q, Zhong M, Wu Y, Zhao L, Zhang F, Cowling BJ, Li F, Leung GM (2020). Author Correction: Temporal dynamics in viral shedding and transmissibility of COVID-19. Nat Med.

[10] He X, Lau EHY, Wu P, Deng X, Wang J, Hao X, Lau YC, Wong JY, Guan Y, Tan X, Mo X, Chen Y, Liao B, Chen W, Hu F, Zhang Q, Zhong M, Wu Y, Zhao L, Zhang F, Cowling BJ, Li F, Leung GM (2020). Temporal dynamics in viral shedding and transmissibility of COVID-19. Nat Med.

[11] Cheng VC, Wong S, Chuang VW, So SY, Chen JH, Sridhar S, To KK, Chan JF, Hung IF, Ho P, Yuen K (2020). The role of community-wide wearing of face mask for control of coronavirus disease 2019 (COVID-19) epidemic due to SARS-CoV-2. J Infect.

[12] Matrajt L, Leung T (2020). Evaluating the effectiveness of social distancing interventions to delay or flatten the epidemic curve of coronavirus disease. Emerg Infect Dis.

[13] Wang Y, Tian H, Zhang L, Zhang M, Guo D, Wu W, Zhang X, Kan GL, Jia L, Huo D, Liu B, Wang X, Sun Y, Wang Q, Yang P, MacIntyre CR (2020). Reduction of secondary transmission of SARS-CoV-2 in households by face mask use, disinfection and social distancing: a cohort study in Beijing, China. BMJ Glob Health.

[14] van Doremalen N, Bushmaker T, Morris DH, Holbrook MG, Gamble A, Williamson BN, Tamin A, Harcourt JL, Thornburg NJ, Gerber SI, Lloyd-Smith JO, de Wit E, Munster VJ (2020). Aerosol and surface stability of SARS-CoV-2 as compared with SARS-CoV-1. N Engl J Med.

[15] Chinazzi M, Davis JT, Ajelli M, Gioannini C, Litvinova M, Merler S, Pastore Y Piontti A, Mu K, Rossi L, Sun K, Viboud C, Xiong X, Yu H, Halloran ME, Longini IM, Vespignani A (2020). The effect of travel restrictions on the spread of the 2019 novel coronavirus (COVID-19) outbreak. Science.

[16] Newsom G (2020). Executive Order N-33-20. California State Government.

[17] Fernandez M (2020). More states issue stay-at-home orders as coronavirus crisis escalates. Axios.

[18] Lyu W, Wehby GL (2020). Comparison of estimated rates of coronavirus disease 2019 (COVID-19) in border counties in Iowa without a stay-at-home order and border counties in Illinois with a stay-at-home order. JAMA Netw Open.

[19] Bonaccorsi G, Pierri F, Cinelli M, Flori A, Galeazzi A, Porcelli F, Schmidt AL, Valensise CM, Scala A, Quattrociocchi W, Pammolli F (2020). Economic and social consequences of human mobility restrictions under COVID-19. Proc Natl Acad Sci U S A.

[20] Community, Work, and School. Centers for Disease Control and Prevention.

[21] Clements JM (2020). Knowledge and behaviors toward COVID-19 among us residents during the early days of the pandemic: cross-sectional online questionnaire. JMIR Public Health Surveill.

[22] Older Adults. Centers for Disease Control and Prevention.

[23] Griffith DM, Sharma G, Holliday CS, Enyia OK, Valliere M, Semlow AR, Stewart EC, Blumenthal RS (2020). Men and COVID-19: A biopsychosocial approach to understanding sex differences in mortality and recommendations for practice and policy interventions. Prev Chronic Dis.

[24] Ortolan A, Lorenzin M, Felicetti M, Doria A, Ramonda R (2020). Does gender influence clinical expression and disease outcomes in COVID-19? A systematic review and meta-analysis. Int J Infect Dis.

[25] Health Equity Considerations and Racial and Ethnic Minority Groups. Centers for Disease Control and Prevention.

[26] Bhopal R (2020). Covid-19: undocumented migrants are probably at greatest risk. BMJ.

[27] Page KR, Venkataramani M, Beyrer C, Polk S (2020). Undocumented U.S. immigrants and Covid-19. N Engl J Med.

[28] Kannan VD, Veazie PJ (2018). Political orientation, political environment, and health behaviors in the United States. Prev Med.

[29] Gostin LO, Friedman EA, Wetter SA (2020). Responding to Covid-19: How to navigate a public health emergency legally and ethically. Hastings Cent Rep.

[30] O'Connor Cathal, Murphy M (2020). Going viral: doctors must tackle fake news in the covid-19 pandemic. BMJ.

[31] Tausanovitch C, Vavreck L, Reny T, Hayes AR, Rudkin A Democracy Fund + UCLA Nationscape Methodology and Representativeness Assessment. Voter Study Group.

[32] (2020). Nationscape Data Set. Voter Study Group.

[33] Hanmer MJ, Kalkan KO (2012). Behind the curve: clarifying the best approach to calculating predicted probabilities and marginal effects from limited dependent variable models. AJPS.

[34] Zaller JR (1992). The Nature and Origins of Mass Opinion.

[35] Hatcher W (2020). President Trump and health care: a content analysis of misleading statements. J Public Health (Oxf).

[36] Dyer O (2020). Trump claims public health warnings on covid-19 are a conspiracy against him. BMJ.

[37] Watson K Coronavirus: How pandemic turned political in Brazil. BBC News.

[38] Shearing H Coronavirus: Why aren't more politicians wearing face masks?. BBC News.

[39] Malecki K, Keating JA, Safdar N (2021). Crisis communication and public perception of COVID-19 risk in the era of social media. Clin Infect Dis.

[40] Rivers C, Martin E, Watson C, Schoch-Spana M, Cicero A, Inglesby T (2020). Resetting our response: changes needed in the US approach to COVID-19. Johns Hopkins Center for Health Security.

[41] Hay SI, IHME COVID-19 Forecasting Team COVID-19 scenarios for the United States. medRxiv..

[42] Howard J, Huang A, Li Z, Tufekci Z Face masks against COVID-19: An evidence review. Preprints..

[43] Wang J, Pan L, Tang S, Ji JS, Shi X (2020). Mask use during COVID-19: A risk adjusted strategy. Environ Pollut.

[44] Ferrer RA, Klein WM (2015). Risk perceptions and health behavior. Curr Opin Psychol.

[45] Weinstein ND, Suls J, Wallston KA (2003). Exploring the Links Between Risk Perceptions and Preventive Health Behavior. Social Foundations of Health and Illness.

[46] Dryhurst S, Schneider CR, Kerr J, Freeman ALJ, Recchia G, van der Bles AM, Spiegelhalter D, van der Linden S (2020). Risk perceptions of COVID-19 around the world. Journal of Risk Research.

[47] Ioannidis JPA (2021). Infection fatality rate of COVID-19 inferred from seroprevalence data. Bull World Health Organ.

[48] Peeling RW, Wedderburn CJ, Garcia PJ, Boeras D, Fongwen N, Nkengasong J, Sall A, Tanuri A, Heymann DL (2020). Serology testing in the COVID-19 pandemic response. Lancet Infect Dis.

[49] Interim Guidelines for COVID-19 Antibody Testing in Clinical and Public Health Settings. Centers for Disease Control and Prevention.

[50] Doshi P (2020). Covid-19: Do many people have pre-existing immunity?. BMJ.

[51] Jena AB, Olenski AR, Khullar D, Bonica A, Rosenthal H (2018). Physicians' political preferences and the delivery of end of life care in the United States: retrospective observational study. BMJ.

[52] Lee TK, Kim HK (2017). Differential effects of message framing on obesity policy support between democrats and republicans. Health Commun.

[53] Hong C (2014). The Politicization of Disease. Public Health Review Internet.

[54] Nyhan B (2014). The Partisan Divide on Ebola Preparedness. New York Times.

[55] Baum MA (2011). Red state, blue state, flu state: media self-selection and partisan gaps in swine flu vaccinations. J Health Polit Policy Law.

[56] Albertson B, Gadarian S (2015). Anxious Politics: Democratic Citizenship in a Threatening World.

[57] Fridman I, Lucas N, Henke D, Zigler CK (2020). Association between public knowledge about COVID-19, trust in information sources, and adherence to social distancing: cross-sectional survey. JMIR Public Health Surveill.

[58] Imburgia TM, Hendrix KS, Donahue KL, Sturm LA, Zimet GD (2017). Predictors of influenza vaccination in the U.S. among children 9-13 years of age. Vaccine.

[59] Kushner Gadarian S, Goodman SW, Pepinsky TB Partisanship, health behavior, and policy attitudes in the early stages of the COVID-19 pandemic. SSRN..

[60] Mesch GS, Schwirian KP (2015). Social and political determinants of vaccine hesitancy: Lessons learned from the H1N1 pandemic of 2009-2010. Am J Infect Control.

[61] Carico RR, Sheppard J, Thomas CB (2021). Community pharmacists and communication in the time of COVID-19: Applying the health belief model. Res Social Adm Pharm.

[62] Bruine de Bruin W, Bennett D (2020). Relationships between initial COVID-19 risk perceptions and protective health behaviors: A national survey. Am J Prev Med.

[63] Bavel JJV, Baicker K, Boggio PS, Capraro V, Cichocka A, Cikara M, Crockett MJ, Crum AJ, Douglas KM, Druckman JN, Drury J, Dube O, Ellemers N, Finkel EJ, Fowler JH, Gelfand M, Han S, Haslam SA, Jetten J, Kitayama S, Mobbs D, Napper LE, Packer DJ, Pennycook G, Peters E, Petty RE, Rand DG, Reicher SD, Schnall S, Shariff A, Skitka LJ, Smith SS, Sunstein CR, Tabri N, Tucker JA, Linden SVD, Lange PV, Weeden KA, Wohl MJA, Zaki J, Zion SR, Willer R (2020). Using social and behavioural science to support COVID-19 pandemic response. Nat Hum Behav.

[64] Wolf MS, Serper M, Opsasnick L, O'Conor RM, Curtis LM, Benavente JY, Wismer G, Batio S, Eifler M, Zheng P, Russell A, Arvanitis M, Ladner D, Kwasny M, Persell SD, Rowe T, Linder JA, Bailey SC (2020). Awareness, attitudes, and actions related to COVID-19 among adults with chronic conditions at the onset of the U.S. outbreak: a cross-sectional survey. Ann Intern Med.

[65] Chowkwanyun M, Reed AL (2020). Racial health disparities and covid-19 - caution and context. N Engl J Med.

[66] Khorram-Manesh A, Carlström E, Hertelendy AJ, Goniewicz K, Casady CB, Burkle FM (2020). Does the prosperity of a country play a role in COVID-19 outcomes?. Disaster Med Public Health Prep.

[67] Parnell A, Goniewicz K, Khorram-Manesh A, Burkle FM, Al-Wathinani A, Hertelendy AJ (2020). COVID-19 a health reform catalyst? —Analyzing single-payer options in the U.S.: Considering economic values, recent proposals, and existing models from abroad. JHA.

[68] The Way Forward on COVID-19: Consensus Guidance on Face Coverings. AAMC.

