# Supplementary material for: Effects of Age, Gender, Health Status, and Political Party on COVID-19–Related Concerns and Prevention Behaviors: Results of a Large, Longitudinal Cross-sectional Survey
Source: JMIR Public Health Surveill. 2021 Apr 28;7(4):e24277. doi: 10.2196/24277 (PMC8080961; doi:10.2196/24277)
Supplement: Multimedia Appendix 1 [file publichealth_v7i4e24277_app1.pdf]

**Appendix 1: CHERRIES Checklist**

| | | |
|---|---|---|
| **Design** | Describe survey design | Target population is general U.S. adult population. Sampling frame includes adult U.S. population. The sample is designed to be representative of the US population, after weighting the respondents. The sample is not a convenience sample, but it is a quota sample derived from a river of respondents. The target weights are derived from the 2018 ACS population over the age of 18, except for 2016 vote, which is derived from teh United States Elections Project and MIT Election Lab. The weights are derived by UCLA Nationscape staff. |
| **IRB (Institutional Review Board) approval and informed consent process** | IRB approval | This study was IRB approved. |
| | Informed consent | Respondents were informed:<br>- The survey was voluntary and that participation could be discontinued at any time<br>- The survey would take about 15 minutes of active participation<br>- They would not be asked for any information that could be used to personally identify them<br>- That any information obtained in connection with this study that could be used to identify them would remain confidential<br>- Of the name and contact information of the corresponding Principal Investigator<br>- That the study was being done to understand people's political views and decisions |
| | Data protection | Respondents were informed that the survey would not ask them for information that could personally identify them -- and that any information obtained in connection with the study that could identify them would remain confidential. |
| **Development and pre-testing** | Development and testing | The survey includes both previously validated items as well as new items developed by the UCLA Nationscape team. The usability and technical functionality of the electronic questionnaire was extensively tested and validated before fielding each survey during a 6 week pilot period prior to fielding. |
| **Recruitment process and description of the sample having access to the questionnaire** | Open survey versus closed survey | This is a closed survey. Respondents are provided by Lucid, a market research platform that runs an online exchange for survey respondents. |
| | Contact mode | Lucid provides the initial contact to the survey respondent online. |
| | Advertising the survey | Survey respondents are provided by Lucid, a market research firm. |
| **Survey administration** | Web/E-mail | This survey is administered online. |
| | Context | This survey was not posted on any webpage. It is taken by invitation only. |
| | Mandatory/voluntary | Not applicable |
| | Incentives | The provision of small incentives vary by the sample provider. |
| | Time/Date | Nationscape fields weekly surveys beginning in the summer of 2019. Survey waves for the analyses presented in this paper were collected between March 19, 2020 and August 5, 2020. |
| | Randomization of items or questionnaires | The order of survey items was not randomized. |
| | Adaptive questioning | Adaptive question was used where appropriate. For example, the question asking independent voters which party they lean towards was not asked to all respondents. |
| | Number of Items | The number of questionnaire items varied by page based on best practices. |
| | Number of screens (pages) | The number of pages varies by survey wave. |
| | Completeness check | Respondents may skip any question they do not want to answer. Don't Know options are offered almost unilaterally. Data validation checks are conducted after the survey is completed to remove those who speed through the survey too quickly or "straightline" responses. |
| | Review step | Respondents were able to review and change their answers. |
| **Response rates** | Unique site visitor | Respondents are provided by a sample provider and are tracked through anonymous unique identifiers. |
| | View rate (Ratio of unique survey visitors/unique site visitors) | Not applicable. |
| | Participation rate (Ratio of unique visitors who agreed to participate/unique first survey page visitors) | The participation rate varies by wave. On average, of those selected to be interviewed, roughly 12% decline immediately and do not proceed. |
| | Completion rate (Ratio of users who finished the survey/users who agreed to participate) | Of those who start the survey, 95% complete the survey. |
| **Preventing multiple entries from the same individual** | Cookies used | Not applicable. Respondents are provided by Lucid. |
| | IP check | Not applicable. Respondents are provided by Lucid. |
| | Log file analysis | Not applicable. Respondents are provided by Lucid. |
| | Registration | Not applicable. Respondents are provided by Lucid. Respondents were able to access the survey after closing the window and did not necessarily complete the survey on the same day they began the survey. |
| **Analysis** | Handling of incomplete questionnaires | Survey respondents who dropped off partway through the survey were excluded. Respondents who opted not to answer certain questions were retained. |
| | Questionnaires submitted with an atypical timestamp | Nationscape fields weekly surveys. Respondents included in a specific wave were both invited to participate and completed the survey in a specific week. |
| | Statistical correction | Nationscape surveys use both a sampling-quota process and a post-stratification weighting process developed by Nationscape staff. For detailed information, see the separate Democracy Fund + UCLA Nationscape Methodology and Representativeness Assessment cited in the article. |
