# Supplementary material for: Effects of Age, Gender, Health Status, and Political Party on COVID-19–Related Concerns and Prevention Behaviors: Results of a Large, Longitudinal Cross-sectional Survey
Source: JMIR Public Health Surveill. 2021 Apr 28;7(4):e24277. doi: 10.2196/24277 (PMC8080961; doi:10.2196/24277)
Supplement: Multimedia Appendix 2 [file publichealth_v7i4e24277_app2.pdf]

Appendix 2: Representativeness Table

Variable	Value	Target %	Unweighted N	Weighted %
Age	18–23	9.4	13653	9.4
	24–29	10.9	13091	10.9
	30–39	17.4	25668	17.4
	40–49	16.3	23494	16.3
	50–59	17	20785	17
	60–69	15.2	19144	15.2
	70+	13.8	9675	13.9
Education	No high school diploma	11.6	11239	11.4
	High school diploma	27.3	27064	27.3
	Some college	21.9	25163	21.9
	Associate's degree	8.4	11239	8.4
	Bachelor's degree	19.3	34497	19.3
	Graduate degree	11.5	16308	11.5
Gender	Male	48.3	58591	48.3
	Female	51.7	66919	51.7
Hispanic	Not Hispanic	83.7	106311	83.8
	Mexican	9.8	11341	9.8
	Other Hispanic	6.5	7858	6.4
Household Income	$19,999 or less	9.7	23317	9.7
	$20,000–$34,999	10.6	18730	10.6
	$35,000–$49,999	10.9	14867	11
	$50,000–$64,999	10.5	12994	10.5
	$65,000–$79,999	9.2	10567	9.2
	$80,000–$99,999	10.4	9081	10.4
	$100,000–$124,999	10.1	10177	10.1
	$125,000–$199,999	14.6	13648	14.6
	$200,000 and above	9.1	5903	9.1
	Unanswered	4.9	6226	4.9

Race	White	74.1	94098	74.1
	Black	12.1	14344	12.1
	AAPI	6.9	7089	6.9
	Other race	7	9979	7
Region	Midwest	20.8	27505	20.7
	Northeast	17.4	23178	17.9
	South	37.9	46515	38
	West	23.8	28312	23.4
Vote in 2016	Clinton	27.7	39425	27.7
	Trump	26.5	43432	26.5
	Other vote	3.3	7584	3.3
	No vote	42.5	35069	42.5
