# Supplementary material for: Effects of Age, Gender, Health Status, and Political Party on COVID-19–Related Concerns and Prevention Behaviors: Results of a Large, Longitudinal Cross-sectional Survey
Source: JMIR Public Health Surveill. 2021 Apr 28;7(4):e24277. doi: 10.2196/24277 (PMC8080961; doi:10.2196/24277)
Supplement: Multimedia Appendix 3 [file publichealth_v7i4e24277_app3.pdf]

Appendix 3: Difference-in-Means Tests

Appendix 3a: Difference in Means over time for willingness to send children to school

Dependent Variable	IV Name	IV Value	Weighted Difference-in-Means
Child to School	Overall	Overall	0.075*** [0.045, 0.104]
	Age	18-39	0.086*** [0.038, 0.134]
		40-64	0.101*** [0.057, 0.145]
		65+	-0.002 [-0.075, 0.071]
	Gender	Female	0.098*** [0.057, 0.139]
		Male	0.05* [0.007, 0.093]
	Party	Democrat	0.026 [-0.018, 0.071]
		Independent	0.06 [-0.019, 0.14]
		Republican	0.136*** [0.091, 0.182]

Appendix 3b: Difference in Means over time for willingness to visit a dentist

Dependent Variable	IV Name	IV Value	Weighted Difference-in-Means
Dentist	Overall	Overall	0.084*** [0.058, 0.111]
	Age	18-39	0.102*** [0.059, 0.146]
		40-64	0.075*** [0.035, 0.115]
		65+	0.073* [0.009, 0.137]
	Gender	Female	0.117*** [0.079, 0.154]
		Male	0.049* [0.011, 0.088]
	Party	Democrat	0.091*** [0.05, 0.132]
		Independent	0.06 [-0.012, 0.132]
		Republican	0.087*** [0.048, 0.127]
