# Supplementary material for: Effects of Age, Gender, Health Status, and Political Party on COVID-19–Related Concerns and Prevention Behaviors: Results of a Large, Longitudinal Cross-sectional Survey
Source: JMIR Public Health Surveill. 2021 Apr 28;7(4):e24277. doi: 10.2196/24277 (PMC8080961; doi:10.2196/24277)
Supplement: Multimedia Appendix 4 [file publichealth_v7i4e24277_app4.pdf]

Appendix 4: Reference categories for regressions

Variable Name	Reference Category
age__bucket	40-64
gender	Female
race__ethnicity__c	White
pid7__collapsed	Democrat
education__c2	Some college
religion__c	Other religious affiliation
is__evangelical	No
household__income__imputed__tercile	HH Income: 2nd Tercile
household__income__missing	Not Missing
extra__sick__you__c	Not Self
extra__sick__family__c	COVID-19: Not Family
extra__sick__work__c	COVID-19: Not Work
extra__sick__other__c	COVID-19: Not Other
extra__prescriptions__3__cat	0-3
news__sources__count	3-6 News Sources
news__sources__social__media__flag	Yes
covid__trend	Late Peak State
wave__int	11
