# Supplementary material for: Effects of Age, Gender, Health Status, and Political Party on COVID-19–Related Concerns and Prevention Behaviors: Results of a Large, Longitudinal Cross-sectional Survey
Source: JMIR Public Health Surveill. 2021 Apr 28;7(4):e24277. doi: 10.2196/24277 (PMC8080961; doi:10.2196/24277)
Supplement: Multimedia Appendix 5 [file publichealth_v7i4e24277_app5.pdf]

| level | Very concerned about coronavirus | Cancelled travel plans for work or pleasure (Precaution) | Not left home for prolonged period? (Precaution) | Bought extra groceries or household supplies (Precaution) | Stopped visiting family or friends? (Precaution) | Washed hands more often? (Precaution) | Worn a mask? (Precaution) |
|---|---|---|---|---|---|---|---|
| | 1.02<br>[0.91, 1.14] | 0.78 ***<br>[0.7, 0.88] | 2.69 ***<br>[2.36, 3.05] | 1.98 ***<br>[1.73, 2.27] | 2.39 ***<br>[2.08, 2.74] | 5.83 ***<br>[4.82, 7.06] | 3.82 ***<br>[2.91, 5.03] |
| 40-64 | 1.52 ***<br>[1.46, 1.59] | 0.89 ***<br>[0.85, 0.93] | 0.82 ***<br>[0.78, 0.86] | 0.75 ***<br>[0.71, 0.8] | 1.15 ***<br>[1.09, 1.21] | 1.2 ***<br>[1.12, 1.29] | 1.37 ***<br>[1.23, 1.53] |
| 65+ | 2.05 ***<br>[1.93, 2.18] | 1.08 *<br>[1.01, 1.14] | 1.18 ***<br>[1.1, 1.26] | 0.67 ***<br>[0.62, 0.72] | 1.6 ***<br>[1.49, 1.73] | 1.81 ***<br>[1.62, 2.02] | 2.38 ***<br>[2.02, 2.8] |
| Late Peak State | 0.83 ***<br>[0.79, 0.87] | 1.1 ***<br>[1.05, 1.16] | 0.93 **<br>[0.88, 0.98] | 0.87 ***<br>[0.82, 0.93] | 0.79 ***<br>[0.74, 0.84] | 0.95<br>[0.87, 1.04] | 0.64 ***<br>[0.55, 0.73] |
| Low Rate | 0.81 ***<br>[0.74, 0.89] | 1.13 **<br>[1.03, 1.24] | 0.95<br>[0.86, 1.05] | 0.89 *<br>[0.79, 0.99] | 0.76 ***<br>[0.68, 0.85] | 1.13<br>[0.96, 1.33] | 0.69 ***<br>[0.54, 0.87] |
| College and above | 1.15 ***<br>[1.1, 1.21] | 1.58 ***<br>[1.5, 1.67] | 1.29 ***<br>[1.21, 1.37] | 1.11 ***<br>[1.04, 1.19] | 1.53 ***<br>[1.43, 1.63] | 1.16 ***<br>[1.06, 1.28] | 1.37 ***<br>[1.19, 1.57] |
| Some college | 0.93 ***<br>[0.89, 0.97] | 1.12 ***<br>[1.07, 1.18] | 1.04<br>[0.99, 1.09] | 0.93 *<br>[0.88, 0.99] | 1.08 **<br>[1.02, 1.14] | 0.9 **<br>[0.84, 0.97] | 0.93<br>[0.83, 1.03] |
| 4+ Prescriptions | 1.47 ***<br>[1.4, 1.54] | 0.92 ***<br>[0.88, 0.97] | 1.2 ***<br>[1.13, 1.27] | 0.98<br>[0.93, 1.04] | 1.02<br>[0.96, 1.08] | 0.98<br>[0.9, 1.07] | 1.36 ***<br>[1.2, 1.55] |
| COVID-19: Family | 1.25 ***<br>[1.13, 1.37] | 1.8 ***<br>[1.61, 2.01] | 1.15 **<br>[1.03, 1.28] | 1.02<br>[0.89, 1.16] | 1.5 ***<br>[1.33, 1.7] | 0.96<br>[0.8, 1.14] | 1.06<br>[0.85, 1.32] |
| COVID-19: Other | 1.29 ***<br>[1.24, 1.35] | 1.61 ***<br>[1.53, 1.7] | 1.52 ***<br>[1.44, 1.61] | 1.19 ***<br>[1.12, 1.26] | 1.98 ***<br>[1.86, 2.12] | 1.7 ***<br>[1.55, 1.86] | 1.86 ***<br>[1.64, 2.11] |
| COVID-19: Work | 1.45 ***<br>[1.36, 1.55] | 1.3 ***<br>[1.21, 1.41] | 0.84 ***<br>[0.78, 0.91] | 1.09 +<br>[0.99, 1.2] | 1.12 **<br>[1.02, 1.23] | 1.24 ***<br>[1.09, 1.42] | 1.46 ***<br>[1.22, 1.74] |
| COVID-19: Self | 1.24 ***<br>[1.12, 1.39] | 0.29 ***<br>[0.26, 0.33] | 0.89 *<br>[0.79, 1] | 1.04<br>[0.89, 1.21] | 0.27 ***<br>[0.24, 0.3] | 1.02<br>[0.84, 1.25] | 0.65 ***<br>[0.52, 0.81] |
| Male | 0.73 ***<br>[0.7, 0.75] | 0.93 ***<br>[0.89, 0.96] | 0.64 ***<br>[0.62, 0.67] | 0.72 ***<br>[0.69, 0.75] | 0.55 ***<br>[0.53, 0.58] | 0.71 ***<br>[0.66, 0.75] | 0.74 ***<br>[0.67, 0.81] |

| | | | | | | | |
|---|---|---|---|---|---|---|---|
| HH Income: $34,999 - $79,999 | 0.97 [0.92, 1.03] | 1.04 [0.99, 1.1] | 1.03 [0.97, 1.09] | 1.19 *** [1.12, 1.27] | 1.26 *** [1.18, 1.34] | 1.28 *** [1.17, 1.4] | 1.43 *** [1.26, 1.63] |
| HH Income: $79,999 and above | 0.95 + [0.9, 1] | 1.33 *** [1.26, 1.4] | 1.17 *** [1.1, 1.24] | 1.28 *** [1.19, 1.36] | 1.44 *** [1.35, 1.53] | 1.18 *** [1.08, 1.29] | 1.5 *** [1.31, 1.71] |
| HH Income Not Missing | 0.94 [0.86, 1.03] | 1.08 [0.98, 1.19] | 1.05 [0.95, 1.17] | 0.94 [0.84, 1.06] | 0.84 *** [0.76, 0.94] | 0.82 ** [0.71, 0.96] | 0.86 [0.69, 1.07] |
| Evangelical | 1.14 *** [1.09, 1.19] | 0.97 [0.93, 1.02] | 0.96 [0.92, 1.01] | 1.11 *** [1.05, 1.17] | 0.86 *** [0.82, 0.91] | 1.14 *** [1.06, 1.23] | 0.8 *** [0.72, 0.89] |
| 3-6 News Sources | 1.49 *** [1.43, 1.56] | 1.38 *** [1.32, 1.45] | 1.33 *** [1.26, 1.39] | 1.46 *** [1.38, 1.54] | 1.41 *** [1.34, 1.49] | 1.85 *** [1.72, 1.99] | 1.56 *** [1.4, 1.73] |
| 7+ News Sources | 2.37 *** [2.23, 2.52] | 2.21 *** [2.07, 2.37] | 1.48 *** [1.38, 1.59] | 2.03 *** [1.88, 2.2] | 1.67 *** [1.55, 1.81] | 2.13 *** [1.91, 2.37] | 2.06 *** [1.75, 2.43] |
| News from Social Media | 1.02 [0.97, 1.06] | 1.21 *** [1.15, 1.26] | 1.13 *** [1.08, 1.19] | 1.28 *** [1.22, 1.35] | 1.22 *** [1.16, 1.29] | 1.19 *** [1.11, 1.28] | 0.95 [0.85, 1.06] |
| Independent | 0.54 *** [0.51, 0.57] | 0.75 *** [0.71, 0.8] | 0.73 *** [0.69, 0.78] | 0.8 *** [0.75, 0.86] | 0.65 *** [0.61, 0.69] | 0.59 *** [0.54, 0.65] | 0.5 *** [0.43, 0.57] |
| Republican | 0.4 *** [0.38, 0.42] | 0.88 *** [0.84, 0.92] | 0.66 *** [0.62, 0.69] | 0.8 *** [0.76, 0.84] | 0.64 *** [0.61, 0.68] | 0.59 *** [0.55, 0.64] | 0.45 *** [0.4, 0.5] |
| Asian or Pacific Islander | 1.48 *** [1.38, 1.59] | 2.05 *** [1.89, 2.23] | 1.43 *** [1.31, 1.56] | 1.51 *** [1.38, 1.66] | 1.3 *** [1.19, 1.43] | 1.25 *** [1.1, 1.42] | 2.1 *** [1.71, 2.58] |
| Black | 1.33 *** [1.25, 1.42] | 1.23 *** [1.15, 1.32] | 0.82 *** [0.77, 0.88] | 1.5 *** [1.38, 1.63] | 0.84 *** [0.78, 0.91] | 0.99 [0.88, 1.11] | 1.22 * [1.03, 1.44] |
| Hispanic | 1.29 *** [1.22, 1.36] | 1.63 *** [1.53, 1.73] | 1.23 *** [1.15, 1.31] | 1.4 *** [1.31, 1.51] | 1.31 *** [1.21, 1.4] | 1.11 * [1, 1.23] | 1.87 *** [1.6, 2.2] |
| Some other race | 1.04 [0.91, 1.18] | 1.26 *** [1.09, 1.45] | 1.03 [0.88, 1.2] | 1.3 *** [1.09, 1.56] | 0.94 [0.79, 1.1] | 0.87 [0.7, 1.07] | 0.88 [0.65, 1.2] |
| Catholic | 1.35 *** [1.25, 1.45] | 1.28 *** [1.19, 1.39] | 0.96 [0.88, 1.05] | 1.07 [0.98, 1.17] | 1.1 * [1, 1.21] | 1.84 *** [1.62, 2.09] | 1.41 *** [1.15, 1.71] |
| Jewish | 1.72 *** [1.51, 1.97] | 1.22 ** [1.06, 1.39] | 1.05 [0.9, 1.22] | 1.51 *** [1.29, 1.78] | 1.14 [0.95, 1.36] | 1.32 * [1.04, 1.67] | 2.54 *** [1.54, 4.2] |
| Mormon | 0.78 ** [0.65, 0.94] | 1.12 [0.93, 1.35] | 0.73 *** [0.6, 0.88] | 0.82 + [0.66, 1.03] | 0.88 [0.72, 1.09] | 0.89 [0.68, 1.16] | 0.73 + [0.52, 1.03] |

| | | | | | | | |
|---|---|---|---|---|---|---|---|
| Other religious affiliation | 1.14 ***<br>[1.07, 1.22] | 1.12 ***<br>[1.04, 1.2] | 0.89 ***<br>[0.82, 0.96] | 1.04<br>[0.96, 1.13] | 0.93<br>[0.86, 1.02] | 1.2 ***<br>[1.07, 1.34] | 1.11<br>[0.93, 1.32] |
| Protestant | 1.04<br>[0.96, 1.12] | 1.14 ***<br>[1.05, 1.24] | 0.96<br>[0.88, 1.05] | 0.96<br>[0.87, 1.05] | 1.07<br>[0.97, 1.19] | 1.37 ***<br>[1.2, 1.57] | 1.23 *<br>[1.01, 1.51] |
| 10 | 0.79 ***<br>[0.73, 0.85] | 1.17 ***<br>[1.08, 1.27] | 1.16 ***<br>[1.07, 1.26] | NA<br>NA | 1.12 **<br>[1.02, 1.23] | 0.76 ***<br>[0.67, 0.87] | 1.16 *<br>[1.03, 1.3] |
| 2 | 1.35 ***<br>[1.25, 1.47] | 1.06<br>[0.97, 1.14] | 1.59 ***<br>[1.46, 1.74] | 0.97<br>[0.89, 1.05] | 1.53 ***<br>[1.4, 1.68] | 1.27 ***<br>[1.09, 1.47] | NA<br>NA |
| 3 | 1.16 ***<br>[1.07, 1.25] | 1.31 ***<br>[1.21, 1.42] | 1.7 ***<br>[1.56, 1.86] | 1.01<br>[0.93, 1.1] | 2.15 ***<br>[1.95, 2.37] | 1.18 *<br>[1.02, 1.36] | NA<br>NA |
| 4 | 1.22 ***<br>[1.13, 1.33] | 1.27 ***<br>[1.17, 1.38] | 1.94 ***<br>[1.77, 2.12] | 1.12 **<br>[1.03, 1.22] | 2.3 ***<br>[2.07, 2.54] | 1.26 ***<br>[1.08, 1.46] | NA<br>NA |
| 5 | 1.06<br>[0.98, 1.15] | 1.25 ***<br>[1.15, 1.36] | 1.73 ***<br>[1.58, 1.89] | 1.1 *<br>[1.01, 1.2] | 1.91 ***<br>[1.73, 2.1] | 1.05<br>[0.91, 1.21] | NA<br>NA |
| 6 | 0.99<br>[0.91, 1.07] | 1.22 ***<br>[1.12, 1.32] | 1.57 ***<br>[1.44, 1.71] | 1.02<br>[0.94, 1.11] | 1.74 ***<br>[1.58, 1.91] | 0.84 **<br>[0.73, 0.96] | NA<br>NA |
| 7 | 0.88 ***<br>[0.81, 0.95] | 1.29 ***<br>[1.19, 1.41] | 1.43 ***<br>[1.31, 1.56] | 1.09 *<br>[1, 1.18] | 1.78 ***<br>[1.62, 1.96] | 0.83 **<br>[0.73, 0.96] | NA<br>NA |
| 8 | 0.87 ***<br>[0.8, 0.95] | 1.23 ***<br>[1.13, 1.35] | 1.31 ***<br>[1.19, 1.44] | NA<br>NA | 1.39 ***<br>[1.25, 1.54] | 0.77 ***<br>[0.66, 0.89] | NA<br>NA |
| 9 | 0.63 ***<br>[0.59, 0.69] | 1.14 ***<br>[1.05, 1.24] | 1.1 *<br>[1.01, 1.2] | NA<br>NA | 1.17 ***<br>[1.07, 1.28] | 0.77 ***<br>[0.68, 0.88] | 1.06<br>[0.94, 1.19] |

## Return Activities (Set 1)

| Variable Level | Send your child to school (Return) | Go to church services (Return) | Go to a stadium concert (Return) | Go to the dentist (Return) | Dinner at friends if safe? (Return) | Fly on an airplane (Return) | Attend a funeral (Return) | Get a haircut (Return) |
|---|---|---|---|---|---|---|---|---|
| | 0.51 ***<br>[0.44, 0.6] | 0.18 ***<br>[0.14, 0.21] | 0.25 ***<br>[0.21, 0.31] | 0.96<br>[0.84, 1.1] | 1.36 ***<br>[1.18, 1.57] | 0.3 ***<br>[0.26, 0.35] | 1<br>[0.87, 1.15] | 0.74 ***<br>[0.64, 0.85] |
| 40-64 | 0.96<br>[0.9, 1.02] | 0.94 +<br>[0.89, 1] | 0.82 ***<br>[0.77, 0.88] | 0.97<br>[0.92, 1.03] | 0.93 **<br>[0.88, 0.98] | 0.87 ***<br>[0.82, 0.92] | 0.9 ***<br>[0.85, 0.95] | 1.01<br>[0.96, 1.07] |
| 65+ | 0.93 +<br>[0.86, 1.01] | 0.94<br>[0.86, 1.02] | 0.41 ***<br>[0.37, 0.46] | 0.88 ***<br>[0.82, 0.95] | 0.78 ***<br>[0.72, 0.84] | 0.75 ***<br>[0.69, 0.81] | 0.66 ***<br>[0.61, 0.71] | 1.01<br>[0.94, 1.09] |
| Late Peak State | 1.01<br>[0.94, 1.08] | 0.89 ***<br>[0.83, 0.95] | 0.87 ***<br>[0.8, 0.93] | 1.1 ***<br>[1.04, 1.17] | 1<br>[0.95, 1.07] | 0.97<br>[0.91, 1.04] | 0.99<br>[0.93, 1.05] | 1.02<br>[0.96, 1.08] |
| Low Rate | 1.1<br>[0.97, 1.25] | 0.97<br>[0.85, 1.1] | 0.86 *<br>[0.74, 1] | 1.39 ***<br>[1.24, 1.56] | 1.25 ***<br>[1.11, 1.41] | 1.02<br>[0.9, 1.16] | 1.12 *<br>[1, 1.26] | 1.02<br>[0.91, 1.15] |
| College and above | 1.19 ***<br>[1.11, 1.28] | 0.99<br>[0.92, 1.06] | 0.8 ***<br>[0.73, 0.86] | 1.11 ***<br>[1.04, 1.19] | 0.98<br>[0.92, 1.05] | 1.11 ***<br>[1.03, 1.19] | 0.81 ***<br>[0.76, 0.86] | 1.01<br>[0.95, 1.08] |
| Some college | 1<br>[0.93, 1.07] | 1.05<br>[0.98, 1.12] | 0.91 **<br>[0.84, 0.97] | 1.09 ***<br>[1.03, 1.16] | 1.1 ***<br>[1.04, 1.17] | 1.01<br>[0.94, 1.07] | 0.98<br>[0.92, 1.04] | 1.02<br>[0.97, 1.09] |
| 4+ Prescriptions | 0.81 ***<br>[0.75, 0.87] | 1<br>[0.93, 1.07] | 0.93 +<br>[0.85, 1.01] | 0.95 +<br>[0.9, 1.01] | 0.83 ***<br>[0.78, 0.88] | 0.82 ***<br>[0.77, 0.88] | 0.96<br>[0.9, 1.02] | 0.93 **<br>[0.87, 0.98] |
| COVID-19: Family | 1.23 ***<br>[1.1, 1.38] | 1.12 +<br>[1, 1.26] | 1.42 ***<br>[1.25, 1.6] | 1.05<br>[0.94, 1.17] | 1.27 ***<br>[1.14, 1.42] | 1.34 ***<br>[1.19, 1.5] | 1.23 ***<br>[1.1, 1.37] | 1.14 *<br>[1.02, 1.27] |
| COVID-19: Other | 0.86 ***<br>[0.81, 0.91] | 0.96<br>[0.9, 1.02] | 0.71 ***<br>[0.67, 0.77] | 0.95 +<br>[0.91, 1.01] | 1.05 +<br>[0.99, 1.11] | 0.84 ***<br>[0.79, 0.89] | 1.02<br>[0.97, 1.08] | 0.92 ***<br>[0.87, 0.97] |
| COVID-19: Work | 1.03<br>[0.95, 1.12] | 1.02<br>[0.94, 1.12] | 1.13 **<br>[1.03, 1.24] | 1.1 **<br>[1.02, 1.19] | 0.97<br>[0.89, 1.05] | 1.16 ***<br>[1.07, 1.26] | 1.01<br>[0.93, 1.09] | 1.12 **<br>[1.03, 1.21] |
| COVID-19: Self | 2.62 ***<br>[2.29, 3] | 1.98 ***<br>[1.72, 2.27] | 3.99 ***<br>[3.48, 4.58] | 1.56 ***<br>[1.36, 1.78] | 1.27 ***<br>[1.11, 1.45] | 2.78 ***<br>[2.44, 3.17] | 1.39 ***<br>[1.22, 1.59] | 1.39 ***<br>[1.22, 1.59] |
| Male | 1.37 ***<br>[1.3, 1.45] | 1.29 ***<br>[1.22, 1.36] | 1.7 ***<br>[1.6, 1.81] | 1.2 ***<br>[1.15, 1.26] | 1.13 ***<br>[1.08, 1.19] | 1.51 ***<br>[1.43, 1.59] | 1.22 ***<br>[1.16, 1.27] | 1.29 ***<br>[1.23, 1.35] |
| | 0.97 | 0.91 * | 0.8 *** | 1.08 * | 1.04 | 0.91 * | 1.03 | 1.15 *** |

| | | | | | | | | |
|---|---|---|---|---|---|---|---|---|
| HH Income: $34,999 - $79,999 | [0.89, 1.05] | [0.84, 0.99] | [0.73, 0.88] | [1.01, 1.16] | [0.97, 1.12] | [0.84, 0.98] | [0.97, 1.11] | [1.07, 1.24] |
| HH Income: $79,999 and above | 1.26 ***<br>[1.16, 1.36] | 0.98<br>[0.91, 1.06] | 0.9 *<br>[0.83, 0.99] | 1.29 ***<br>[1.2, 1.38] | 1.16 ***<br>[1.08, 1.24] | 1.19 ***<br>[1.1, 1.28] | 1.25 ***<br>[1.17, 1.35] | 1.4 ***<br>[1.3, 1.5] |
| HH Income Not Missing | 0.97<br>[0.85, 1.11] | 1.22 ***<br>[1.08, 1.39] | 1.02<br>[0.88, 1.18] | 1.02<br>[0.91, 1.14] | 1.05<br>[0.94, 1.18] | 1.13 +<br>[1, 1.29] | 1.12 *<br>[1, 1.26] | 1.05<br>[0.94, 1.17] |
| Evangelical | 1.18 ***<br>[1.11, 1.26] | 2.07 ***<br>[1.95, 2.19] | 1.5 ***<br>[1.4, 1.6] | 0.93 **<br>[0.88, 0.98] | 0.99<br>[0.93, 1.05] | 1.21 ***<br>[1.14, 1.29] | 1.13 ***<br>[1.06, 1.19] | 1.07 *<br>[1.01, 1.13] |
| 3-6 News Sources | 0.83 ***<br>[0.78, 0.89] | 0.85 ***<br>[0.8, 0.91] | 0.84 ***<br>[0.78, 0.91] | 0.92 ***<br>[0.87, 0.97] | 0.99<br>[0.93, 1.05] | 0.88 ***<br>[0.82, 0.94] | 0.92 ***<br>[0.87, 0.97] | 0.97<br>[0.91, 1.02] |
| 7+ News Sources | 0.9 *<br>[0.83, 0.98] | 0.93<br>[0.86, 1.02] | 1.14 **<br>[1.03, 1.25] | 0.92 *<br>[0.85, 0.99] | 0.84 ***<br>[0.78, 0.91] | 0.99<br>[0.91, 1.08] | 0.87 ***<br>[0.8, 0.94] | 0.89 ***<br>[0.82, 0.96] |
| News from Social Media | 1.03<br>[0.97, 1.1] | 1.11 ***<br>[1.05, 1.18] | 1.17 ***<br>[1.09, 1.26] | 0.96<br>[0.91, 1.02] | 1.16 ***<br>[1.1, 1.23] | 1.15 ***<br>[1.08, 1.22] | 1.12 ***<br>[1.06, 1.19] | 1.08 **<br>[1.02, 1.15] |
| Independent | 1.21 ***<br>[1.11, 1.31] | 1.26 ***<br>[1.17, 1.37] | 1.57 ***<br>[1.43, 1.72] | 1.14 ***<br>[1.06, 1.22] | 1.3 ***<br>[1.21, 1.39] | 1.27 ***<br>[1.18, 1.38] | 1.23 ***<br>[1.14, 1.32] | 1.28 ***<br>[1.19, 1.37] |
| Republican | 1.7 ***<br>[1.6, 1.81] | 2.22 ***<br>[2.09, 2.36] | 2 ***<br>[1.87, 2.15] | 1.54 ***<br>[1.46, 1.63] | 1.82 ***<br>[1.72, 1.92] | 1.77 ***<br>[1.67, 1.88] | 1.7 ***<br>[1.61, 1.79] | 1.78 ***<br>[1.68, 1.88] |
| Asian or Pacific Islander | 0.86 ***<br>[0.78, 0.95] | 0.68 ***<br>[0.61, 0.75] | 0.57 ***<br>[0.51, 0.64] | 0.58 ***<br>[0.54, 0.64] | 0.58 ***<br>[0.53, 0.63] | 0.84 ***<br>[0.76, 0.92] | 0.57 ***<br>[0.52, 0.62] | 0.61 ***<br>[0.56, 0.66] |
| Black | 0.74 ***<br>[0.67, 0.81] | 1.01<br>[0.92, 1.1] | 0.87 **<br>[0.78, 0.97] | 0.71 ***<br>[0.66, 0.77] | 0.52 ***<br>[0.48, 0.57] | 0.93<br>[0.85, 1.02] | 0.58 ***<br>[0.54, 0.63] | 0.53 ***<br>[0.49, 0.58] |
| Hispanic | 0.81 ***<br>[0.74, 0.87] | 0.71 ***<br>[0.66, 0.77] | 0.81 ***<br>[0.74, 0.89] | 0.79 ***<br>[0.73, 0.85] | 0.68 ***<br>[0.63, 0.73] | 0.87 ***<br>[0.81, 0.95] | 0.56 ***<br>[0.53, 0.61] | 0.75 ***<br>[0.7, 0.81] |
| Some other race | 0.76 **<br>[0.62, 0.93] | 0.7 ***<br>[0.57, 0.87] | 0.68 ***<br>[0.54, 0.86] | 0.73 ***<br>[0.62, 0.86] | 0.65 ***<br>[0.55, 0.77] | 0.97<br>[0.81, 1.17] | 0.64 ***<br>[0.54, 0.76] | 0.72 ***<br>[0.61, 0.86] |
| Catholic | 0.97<br>[0.87, 1.09] | 3.36 ***<br>[2.87, 3.95] | 1.14 *<br>[1.01, 1.3] | 1.05<br>[0.96, 1.15] | 0.9 *<br>[0.82, 0.99] | 1<br>[0.9, 1.11] | 1.19 ***<br>[1.08, 1.3] | 1.24 ***<br>[1.13, 1.36] |
| Jewish | 1.04<br>[0.86, 1.26] | 2.16 ***<br>[1.73, 2.7] | 1.26 *<br>[1.02, 1.57] | 1.13<br>[0.96, 1.32] | 0.89<br>[0.76, 1.04] | 1.05<br>[0.88, 1.25] | 1.14<br>[0.97, 1.33] | 1.1<br>[0.93, 1.29] |
| Mormon | 1.31 *<br>[1.02, 1.68] | 6.31 ***<br>[4.82, 8.25] | 0.88<br>[0.66, 1.18] | 1.41 ***<br>[1.11, 1.78] | 1.29 *<br>[1.01, 1.66] | 0.98<br>[0.77, 1.25] | 1.73 ***<br>[1.37, 2.19] | 1.29 *<br>[1.02, 1.63] |
| | 0.85 *** | 2.58 *** | 1.19 ** | 1.04 | 0.95 | 1.02 | 1.13 ** | 1.16 *** |

| | | | | | | | | |
|---|---|---|---|---|---|---|---|---|
| Other religious affiliation | [0.76, 0.94] | [2.21, 3.02] | [1.05, 1.33] | [0.95, 1.13] | [0.87, 1.04] | [0.92, 1.12] | [1.04, 1.24] | [1.06, 1.27] |
| Protestant | 0.97<br>[0.86, 1.09] | 3.25 ***<br>[2.75, 3.82] | 0.97<br>[0.85, 1.12] | 1.15 **<br>[1.04, 1.27] | 1.09<br>[0.98, 1.2] | 0.85 **<br>[0.76, 0.95] | 1.24 ***<br>[1.12, 1.37] | 1.35 ***<br>[1.22, 1.49] |
| 10 | 0.93<br>[0.85, 1.02] | 1.02<br>[0.93, 1.11] | 1<br>[0.9, 1.1] | 1.16 ***<br>[1.07, 1.25] | 1.04<br>[0.96, 1.13] | 1.01<br>[0.92, 1.1] | 0.87 ***<br>[0.81, 0.95] | 1.1 *<br>[1.02, 1.2] |
| 6 | 0.91 *<br>[0.83, 0.99] | 0.87 ***<br>[0.79, 0.95] | 0.82 ***<br>[0.74, 0.91] | 0.97<br>[0.9, 1.05] | 0.93 +<br>[0.86, 1.01] | 0.94<br>[0.86, 1.02] | 0.84 ***<br>[0.78, 0.91] | 0.94<br>[0.87, 1.02] |
| 7 | 0.85 ***<br>[0.78, 0.93] | 0.88 **<br>[0.81, 0.96] | 0.96<br>[0.87, 1.07] | 0.97<br>[0.89, 1.04] | 0.95<br>[0.88, 1.03] | 1.01<br>[0.93, 1.11] | 0.88 ***<br>[0.81, 0.95] | 0.95<br>[0.87, 1.02] |
| 8 | 0.95<br>[0.87, 1.05] | 0.95<br>[0.86, 1.04] | 1.04<br>[0.94, 1.16] | 1.08 +<br>[0.99, 1.18] | 0.94<br>[0.86, 1.03] | 1.12 *<br>[1.02, 1.23] | 0.9 *<br>[0.83, 0.98] | 0.94<br>[0.86, 1.03] |
| 9 | 1.02<br>[0.93, 1.11] | 0.95<br>[0.87, 1.04] | 0.94<br>[0.85, 1.04] | 1.11 **<br>[1.02, 1.2] | 1.02<br>[0.94, 1.1] | 1.03<br>[0.94, 1.12] | 0.94<br>[0.87, 1.02] | 1.04<br>[0.96, 1.12] |

## Return Activities (Set 2)

| Variable Level | Go to the movies (Return) | Ride on public transportation (Return) | Eat at restaurant if safe? (Return) | Return to school yourself (Return) | Shop if safe? (Return) | Attend a professional sporting event (Return) | Attend a wedding reception (Return) |
|---|---|---|---|---|---|---|---|
| | 0.39 ***<br>[0.33, 0.46] | 0.4 ***<br>[0.34, 0.48] | 0.83 **<br>[0.73, 0.95] | 0.52 ***<br>[0.44, 0.61] | 0.49 ***<br>[0.42, 0.56] | 0.21 ***<br>[0.17, 0.25] | 0.52 ***<br>[0.45, 0.6] |
| 40-64 | 0.8 ***<br>[0.75, 0.85] | 0.89 ***<br>[0.83, 0.95] | 0.88 ***<br>[0.83, 0.93] | 0.68 ***<br>[0.64, 0.73] | 0.9 ***<br>[0.85, 0.95] | 0.83 ***<br>[0.77, 0.88] | 0.84 ***<br>[0.8, 0.89] |
| 65+ | 0.54 ***<br>[0.49, 0.59] | 0.62 ***<br>[0.56, 0.68] | 0.73 ***<br>[0.68, 0.79] | 0.48 ***<br>[0.44, 0.53] | 0.81 ***<br>[0.75, 0.87] | 0.47 ***<br>[0.42, 0.52] | 0.67 ***<br>[0.63, 0.73] |
| Late Peak State | 1.01<br>[0.94, 1.08] | 0.72 ***<br>[0.67, 0.78] | 0.99<br>[0.94, 1.05] | 1.01<br>[0.94, 1.08] | 0.97<br>[0.91, 1.03] | 0.9 **<br>[0.84, 0.97] | 0.93 *<br>[0.88, 0.99] |
| Low Rate | 1.01<br>[0.89, 1.15] | 0.78 ***<br>[0.67, 0.89] | 0.98<br>[0.87, 1.1] | 1.14 +<br>[0.99, 1.31] | 1.06<br>[0.95, 1.19] | 0.96<br>[0.83, 1.11] | 1.07<br>[0.96, 1.21] |
| College and above | 0.77 ***<br>[0.72, 0.83] | 0.93 +<br>[0.85, 1] | 0.89 ***<br>[0.83, 0.95] | 1.03<br>[0.95, 1.11] | 0.89 ***<br>[0.83, 0.95] | 0.83 ***<br>[0.76, 0.9] | 0.96<br>[0.9, 1.02] |
| Some college | 0.87 ***<br>[0.82, 0.93] | 0.88 ***<br>[0.81, 0.94] | 0.97<br>[0.92, 1.03] | 1.05<br>[0.98, 1.13] | 0.97<br>[0.92, 1.03] | 0.93 +<br>[0.87, 1] | 0.98<br>[0.92, 1.04] |
| 4+ Prescriptions | 0.9 ***<br>[0.84, 0.97] | 0.8 ***<br>[0.74, 0.87] | 0.94 *<br>[0.89, 1] | 0.75 ***<br>[0.69, 0.81] | 0.95 +<br>[0.89, 1.01] | 0.89 ***<br>[0.82, 0.96] | 0.95 +<br>[0.89, 1.01] |
| COVID-19: Family | 1.42 ***<br>[1.26, 1.59] | 1.5 ***<br>[1.34, 1.69] | 1.3 ***<br>[1.17, 1.44] | 1.34 ***<br>[1.19, 1.51] | 1.28 ***<br>[1.15, 1.43] | 1.35 ***<br>[1.19, 1.53] | 1.34 ***<br>[1.21, 1.5] |
| COVID-19: Other | 0.81 ***<br>[0.76, 0.86] | 0.77 ***<br>[0.72, 0.83] | 0.85 ***<br>[0.8, 0.89] | 0.93 *<br>[0.87, 0.99] | 0.74 ***<br>[0.7, 0.78] | 0.76 ***<br>[0.71, 0.81] | 0.89 ***<br>[0.85, 0.94] |
| COVID-19: Work | 1.05<br>[0.97, 1.14] | 1.16 ***<br>[1.06, 1.27] | 1.02<br>[0.95, 1.1] | 0.99<br>[0.9, 1.08] | 1.07 +<br>[0.99, 1.16] | 1.08 +<br>[0.99, 1.19] | 1.05<br>[0.97, 1.13] |
| COVID-19: Self | 2.88 ***<br>[2.52, 3.28] | 3.09 ***<br>[2.7, 3.54] | 1.81 ***<br>[1.59, 2.06] | 2.57 ***<br>[2.24, 2.95] | 2.36 ***<br>[2.07, 2.68] | 3.52 ***<br>[3.07, 4.04] | 1.99 ***<br>[1.75, 2.26] |
| Male | 1.59 ***<br>[1.51, 1.68] | 1.89 ***<br>[1.78, 2] | 1.44 ***<br>[1.37, 1.51] | 1.31 ***<br>[1.23, 1.38] | 1.51 ***<br>[1.44, 1.58] | 2 ***<br>[1.88, 2.12] | 1.28 ***<br>[1.22, 1.34] |

| | | | | | | | |
|---|---|---|---|---|---|---|---|
| HH Income: $34,999 - $79,999 | 0.93 +<br>[0.86, 1] | 0.65 ***<br>[0.59, 0.7] | 0.94 +<br>[0.88, 1.01] | 0.87 ***<br>[0.8, 0.94] | 0.96<br>[0.89, 1.03] | 0.85 ***<br>[0.78, 0.93] | 1.03<br>[0.96, 1.11] |
| HH Income: $79,999 and above | 1.05<br>[0.97, 1.14] | 0.73 ***<br>[0.67, 0.79] | 1.08 *<br>[1, 1.15] | 1.21 ***<br>[1.11, 1.31] | 1.02<br>[0.95, 1.09] | 1.01<br>[0.92, 1.1] | 1.14 ***<br>[1.06, 1.22] |
| HH Income Not Missing | 1.1<br>[0.97, 1.24] | 1.14 +<br>[0.99, 1.32] | 1.04<br>[0.93, 1.16] | 1.25 ***<br>[1.1, 1.43] | 1.05<br>[0.94, 1.18] | 1.06<br>[0.91, 1.23] | 0.96<br>[0.85, 1.08] |
| Evangelical | 1.37 ***<br>[1.29, 1.46] | 1.4 ***<br>[1.31, 1.5] | 1.21 ***<br>[1.15, 1.28] | 1.2 ***<br>[1.13, 1.29] | 1.27 ***<br>[1.2, 1.35] | 1.36 ***<br>[1.28, 1.46] | 1.24 ***<br>[1.17, 1.32] |
| 3-6 News Sources | 0.78 ***<br>[0.73, 0.83] | 0.93 +<br>[0.86, 1] | 0.87 ***<br>[0.82, 0.92] | 0.96<br>[0.89, 1.03] | 0.92 **<br>[0.87, 0.98] | 0.82 ***<br>[0.76, 0.88] | 0.82 ***<br>[0.77, 0.87] |
| 7+ News Sources | 0.98<br>[0.9, 1.06] | 1.35 ***<br>[1.23, 1.48] | 0.88 ***<br>[0.82, 0.95] | 0.99<br>[0.91, 1.09] | 1.12 ***<br>[1.04, 1.21] | 1.09 +<br>[0.99, 1.19] | 0.87 ***<br>[0.81, 0.95] |
| News from Social Media | 1.14 ***<br>[1.07, 1.22] | 1.07 +<br>[1, 1.15] | 1.07 **<br>[1.02, 1.13] | 1.21 ***<br>[1.12, 1.29] | 1.06 *<br>[1, 1.12] | 1.23 ***<br>[1.15, 1.32] | 1.14 ***<br>[1.07, 1.2] |
| Independent | 1.42 ***<br>[1.31, 1.54] | 1.25 ***<br>[1.15, 1.37] | 1.37 ***<br>[1.28, 1.47] | 1.05<br>[0.96, 1.15] | 1.32 ***<br>[1.22, 1.41] | 1.41 ***<br>[1.29, 1.55] | 1.36 ***<br>[1.26, 1.46] |
| Republican | 1.79 ***<br>[1.69, 1.9] | 1.55 ***<br>[1.45, 1.66] | 2.03 ***<br>[1.92, 2.14] | 1.55 ***<br>[1.46, 1.66] | 1.8 ***<br>[1.71, 1.9] | 1.96 ***<br>[1.83, 2.09] | 2.02 ***<br>[1.91, 2.13] |
| Asian or Pacific Islander | 0.65 ***<br>[0.59, 0.73] | 0.86 **<br>[0.77, 0.96] | 0.75 ***<br>[0.68, 0.81] | 0.87 **<br>[0.78, 0.96] | 0.88 ***<br>[0.8, 0.96] | 0.52 ***<br>[0.46, 0.59] | 0.64 ***<br>[0.59, 0.71] |
| Black | 0.86 ***<br>[0.78, 0.94] | 1.27 ***<br>[1.16, 1.4] | 0.66 ***<br>[0.6, 0.71] | 0.8 ***<br>[0.73, 0.88] | 0.93 +<br>[0.85, 1.01] | 0.9 *<br>[0.81, 1] | 0.73 ***<br>[0.67, 0.8] |
| Hispanic | 0.82 ***<br>[0.75, 0.88] | 1.13 **<br>[1.04, 1.23] | 0.72 ***<br>[0.67, 0.77] | 0.86 ***<br>[0.8, 0.94] | 0.87 ***<br>[0.81, 0.94] | 0.81 ***<br>[0.74, 0.88] | 0.61 ***<br>[0.57, 0.66] |
| Some other race | 0.83 +<br>[0.68, 1.01] | 1.2 +<br>[0.98, 1.48] | 0.81 **<br>[0.68, 0.96] | 0.77 **<br>[0.63, 0.95] | 1.02<br>[0.86, 1.21] | 0.75 **<br>[0.6, 0.94] | 0.78 **<br>[0.65, 0.93] |
| Catholic | 1.07<br>[0.96, 1.19] | 0.87 *<br>[0.77, 0.98] | 0.97<br>[0.89, 1.07] | 0.92<br>[0.82, 1.03] | 1.05<br>[0.95, 1.15] | 1.41 ***<br>[1.24, 1.61] | 1.23 ***<br>[1.11, 1.35] |
| Jewish | 1<br>[0.83, 1.21] | 0.95<br>[0.77, 1.17] | 0.84 *<br>[0.72, 0.98] | 1.11<br>[0.9, 1.36] | 0.92<br>[0.78, 1.08] | 1.37 ***<br>[1.1, 1.69] | 1.13<br>[0.96, 1.33] |
| Mormon | 1.1<br>[0.86, 1.41] | 1.19<br>[0.91, 1.56] | 0.98<br>[0.79, 1.22] | 0.94<br>[0.72, 1.22] | 1.12<br>[0.89, 1.4] | 1.11<br>[0.84, 1.47] | 1.96 ***<br>[1.56, 2.46] |

| | | | | | | | |
|---|---|---|---|---|---|---|---|
| Other religious affiliation | 1.06<br>[0.96, 1.17] | 0.94<br>[0.84, 1.05] | 0.94<br>[0.86, 1.02] | 0.84 ***<br>[0.76, 0.94] | 1.03<br>[0.94, 1.13] | 1.28 ***<br>[1.14, 1.45] | 1.13 **<br>[1.03, 1.24] |
| Protestant | 1.04<br>[0.93, 1.17] | 0.87 *<br>[0.76, 0.99] | 0.98<br>[0.89, 1.08] | 0.94<br>[0.83, 1.06] | 0.99<br>[0.89, 1.1] | 1.28 ***<br>[1.11, 1.46] | 1.2 ***<br>[1.08, 1.33] |
| 10 | 0.94<br>[0.86, 1.03] | 1.07<br>[0.97, 1.18] | 0.83 ***<br>[0.77, 0.9] | 1.14 **<br>[1.04, 1.25] | 1.07 +<br>[0.99, 1.16] | 0.97<br>[0.88, 1.07] | 0.94<br>[0.86, 1.01] |
| 6 | 0.9 **<br>[0.82, 0.98] | 1<br>[0.9, 1.1] | 0.84 ***<br>[0.78, 0.91] | 0.94<br>[0.86, 1.04] | 0.84 ***<br>[0.77, 0.91] | 0.82 ***<br>[0.74, 0.9] | 0.87 ***<br>[0.8, 0.94] |
| 7 | 0.97<br>[0.89, 1.06] | 1.02<br>[0.93, 1.13] | 0.81 ***<br>[0.75, 0.88] | 0.95<br>[0.86, 1.04] | 0.95<br>[0.88, 1.03] | 1.01<br>[0.91, 1.11] | 0.95<br>[0.88, 1.03] |
| 8 | 0.94<br>[0.85, 1.03] | 0.99<br>[0.89, 1.11] | 0.84 ***<br>[0.77, 0.92] | 0.99<br>[0.89, 1.1] | 0.97<br>[0.89, 1.06] | 1.01<br>[0.91, 1.12] | 0.97<br>[0.89, 1.06] |
| 9 | 0.95<br>[0.87, 1.04] | 1.03<br>[0.94, 1.14] | 0.88 ***<br>[0.81, 0.95] | 1.17 ***<br>[1.06, 1.29] | 1.02<br>[0.94, 1.1] | 1.02<br>[0.92, 1.12] | 1<br>[0.92, 1.08] |
